# Mass Spectral Molecular Networking to Profile the Metabolome of Biostimulant *Bacillus* Strains

**DOI:** 10.3389/fpls.2022.920963

**Published:** 2022-06-09

**Authors:** Lerato Nephali, Paul Steenkamp, Karl Burgess, Johan Huyser, Margaretha Brand, Justin J. J. van der Hooft, Fidele Tugizimana

**Affiliations:** ^1^Department of Biochemistry, University of Johannesburg, Johannesburg, South Africa; ^2^School of Biological Sciences, Institute of Quantitative Biology, Biochemistry, and Biotechnology, The University of Edinburgh, Edinburgh, United Kingdom; ^3^International Research and Development Division, Omnia Group, Ltd., Johannesburg, South Africa; ^4^Bioinformatics Group, Wageningen University, Wageningen, Netherlands

**Keywords:** *Bacillus*, biostimulants, GNPS platform, metabolomics, molecular networking, lipopeptides

## Abstract

Beneficial soil microbes like plant growth-promoting rhizobacteria (PGPR) significantly contribute to plant growth and development through various mechanisms activated by plant-PGPR interactions. However, a complete understanding of the biochemistry of the PGPR and microbial intraspecific interactions within the consortia is still enigmatic. Such complexities constrain the design and use of PGPR formulations for sustainable agriculture. Therefore, we report the application of mass spectrometry (MS)-based untargeted metabolomics and molecular networking (MN) to interrogate and profile the intracellular chemical space of PGPR *Bacillus* strains: *B. laterosporus*, *B. amyloliquefaciens*, *B. licheniformis* 1001, and *B. licheniformis* M017 and their consortium. The results revealed differential and diverse chemistries in the four *Bacillus* strains when grown separately, and also differing from when grown as a consortium. MolNetEnhancer networks revealed 11 differential molecular families that are comprised of lipids and lipid-like molecules, benzenoids, nucleotide-like molecules, and organic acids and derivatives. Consortium and *B. amyloliquefaciens* metabolite profiles were characterized by the high abundance of surfactins, whereas *B. licheniformis* strains were characterized by the unique presence of lichenysins. Thus, this work, applying metabolome mining tools, maps the microbial chemical space of isolates and their consortium, thus providing valuable insights into molecular information of microbial systems. Such fundamental knowledge is essential for the innovative design and use of PGPR-based biostimulants.

## Introduction

*Bacillus* spp. are plant growth-promoting rhizobacteria (PGPR)—part of the “core plant root microbiome” communities that are ubiquitous across a wide range of environments and host species. The belowground plant-PGPR interactions are established in a highly dynamic, sophisticated, and controlled manner through a wide range of specialized metabolites and involve reprogramming of gene expression in either one or both of the interacting partners (Sasse et al., 2018). This mutual chemical communication alters plant growth, nutrient availability, inhibition of soil pathogens, enhanced plant defenses, biofilm development, and recruitment of other soil microbes (Vandenkoornhuyse et al., 2015). Untangling these chemical intercommunications is still inscrutable and subject to ongoing research. One of the approaches is to characterize metabolic profiles, i.e., chemistries of different partners, such as *Bacillus* spp. in this case, or the plant roots (Nguyen et al., 2013; Korenblum et al., 2020; Shahid et al., 2021; Sun et al., 2022). Such efforts would reveal not only a global metabolic map, but also the key chemical repertoires, potential drivers of the plant-PGPR interactions, contributing also toward an innovative design and formulation of PGPR-based biostimulants.

Thus, to interrogate the chemical capabilities of PGPR, this study employs untargeted liquid chromatography-mass spectrometry (LC-MS)-based metabolomics that generate tandem mass (MS/MS) spectra, and applies molecular networking (MN) approaches to decode and characterize the chemical space of four *Bacillus* isolates and a consortium of *Bacillus* strains. Metabolomics is a multidisciplinary *omics* science that allows the quantitative and qualitative evaluation of all measurable metabolites, constituting a chemical space (the metabolome) that carries both genetic and environmental imprints (Tugizimana et al., 2013; Courant et al., 2014). Compared to other -ome levels (i.e., genome, transcriptome, and proteome) in systems biology, the metabolome is more sensitive to perturbations and is regarded as the ultimate expression of all activities of metabolic pathways. Moreover, fluctuations in the metabolome are amplified relative to changes in the transcriptome and proteome, and are arguably numerically more tractable (Kell et al., 2005).

Mass spectral mining strategies such as molecular networking (MN) and MS2LDA substructure discovery are rapidly gaining popularity in untargeted metabolomics due to the advantageous ability to provide a comprehensive visualization of the chemistries within various biological systems and the easy link to annotation tools that offer automated mass spectral annotation (da Silva et al., 2018; Rogers et al., 2019). MN employs a vector-based computational algorithm to compare and weigh the degree of spectral similarity of large mass spectrometry (MS) dataset, on the basis that structurally related molecules will yield similar fragmentation patterns. Similar MS/MS spectra are organized and presented in graph-based spectral networks allowing the global visualization of all untargeted chemical signatures (known and unknown chemical repertoires) detected by the mass spectrometer (Quinn et al., 2017; Fox Ramos et al., 2019; Beniddir et al., 2021). Furthermore, MN offers unique advantages, such as the ability to decode the metabolomics “dark matter” (Quinn et al., 2017). Thus, the work presented herein applies MN strategies in annotating the metabolome of *Bacillus*, providing insights into the molecular space of the bacteria. Our efforts advance the understanding of *Bacillus* spp.’s metabolome, both as isolates and as a consortium, generating thus fundamental knowledgebase necessary for industries to confidently and innovatively explore and design PGPR-based formulations. In turn, this would provide a great impetus in implementing PGPR-based strategies into agronomic practices for sustainable agriculture and food production.

## Materials and Methods

All chemicals for sample analyses (from the pre-analytical step to the data acquisition stage) were of pure-grade quality and obtained from various international suppliers. Briefly, the organic solvents used, methanol and acetonitrile, were LC-MS grade quality (Romil, SPS, Cambridge, United Kingdom). Water was purified by a Milli-Q Gradient A10 system (Siemens, Fahrenburg, Germany). Leucine enkephalin and formic acid were purchased from Sigma Aldrich, Munich, Germany. Four bacterial isolates selected for this study belong to the *Bacillus* genus, the species included *B. licheniformis* M017, *B. licheniformis* 1001, *B. amyloliquefaciens*, and *B. laterosporus* strains. The microbial consortium is a commercial product—Bacstim^®^ 100, containing equal volumes of the above-mentioned *Bacillus* strains (Omnia Group Ltd., South Africa). The biostimulant activity of this microbial consortium, Bacstim^®^ 100, has been tested on maize plants under greenhouse conditions, where enhanced plant growth and drought tolerance was demonstrated (Nephali et al., 2021; Othibeng et al., 2022).

### Bacterial Culturing, Harvesting, and Metabolite Extraction

To monitor the growth rates of bacteria, 100 μL of bacterial stocks (isolates—*B. licheniformis* M017, *B. licheniformis* 1001, *B. amyloliquefaciens*, and *B. laterosporus* and the consortium, respectively) were inoculated into 20 mL Lysogeny broth (LB) media under sterile conditions and incubated at 28°C in a shaker at 140 rpm for 36 h. The OD_600_ readings of the bacterial cultures were taken every 3 h to determine the bacterial growth curves (Supplementary Figure 1). From the growth curves, 4-time points were selected for harvesting and metabolite extraction: 3 h (lag phase), 7.5 h (exponential phase), 24 and 31.5 h (stationary phase) (Supplementary Figure 1). This growth curve was then used for bacterial cell harvesting. Following the conditions mentioned above, culturing flasks were prepared in triplicates for *Bacillus* isolates and consortium. At the selected time points (i.e., the determined growth phases), the cells were harvested by centrifuging the cultures at 5,000 rpm at 28°C for 15 min. Thereafter, the supernatants were decanted into new 50 mL falcon tubes and the wet weight of pellets was measured. Pellets were stored at −80°C until the metabolite extraction. The intracellular metabolites were extracted by adding 100% LC-MS grade methanol to the pellet (15 mg: 320 μL). The mixture was then vortexed, followed by sonication with a probe sonicator at 55% for 1 min. The homogenous mixtures were centrifuged at 3,700 rpm for 30 min at 28°C. The supernatants (intracellular metabolites samples) were filtered into pre-labeled HPLC glass vials fitted with 500 μL inserts (Shimadzu, South Africa) using 0.22 μm nylon filters and 1 mL syringes.

### Sample Analysis—Data Acquisition

Intracellular extracts were analyzed on an Acquity UHPLC system hyphenated with SYNAPT G1 high definition quadrupole time-of-flight mass spectrometer (HD-Q-TOF-MS) equipped with an electrospray ionization (ESI) source (Waters Corporations, Milford, United States). Chromatographic separation was performed on an analytical C18 column HSS T3 (150 mm × 2.1 mm, 1.7 μm) with an injection volume of 3 μL. A binary solvent system was used as mobile phase, solvent (A) consisting of ultra-clear milli-Q water with 0.1% (v/v) formic acid; solvent (B) consisting of 100% acetonitrile with 0.1% (v/v) formic acid. The gradient elution was carried out at a constant flow rate of 0.4 mL min^–1^, using the following conditions: initial conditions of 99% (solvent A) and 1% (solvent B) for 1 min. Solvent B was then gradually increased to 99% from 1–24 min and held constant for 3 min. From 27 to 28 min, solvent B was increased to 100% and held constant until 30 min (end of run). For mass spectrometry analyses, ionization was carried out in ESI positive mode with a 50–1,200 Da, and 0.2 s scan range and scan time, respectively. Other MS parameters were set as follows: source temperature of 120°C, desolvation temperature of 450°C, 2.5 kV capillary voltage, with 17 and 4 V sampling and extraction cone voltages, respectively, at a desolvation gas flow rate of 550 L/h. Both full-scan (unfragemented) and fragmentation MS analyses were carried out. A data independent acquisition (DIA) method, MS*^E^*, was applied (with collision energy ramping from 10 to 40 eV) to obtained fragmentation spectral data.

### Molecular Networking in the Global Natural Product Social Platform

The acquired (ESI positive) mass spectral data files (.raw Waters) were converted to Mass Spectrometry-Data Independent AnaLysis (MS-DIAL) compatible formats using the Reifys Abf (analysis base file) converter software.^1^ The ABF files were then uploaded onto the MS-DIAL platform for data-processing and mass spectral deconvolution of data-independent acquisition (DIA). The data processing parameters used in MS-DIAL were as follows: mass accuracy (MS1 and MS2 tolerance) was set at 0.05 Da, the minimum peak height was set at 10 amplitude, mass slice width set at 0.1 Da for peak detection, a sigma window value set at 0.5 and retention time tolerance set at 0.2 min. The acquired peak spots (features) after processing were 543, 567, 603, 3,899, and 2,983 for consortium, *B. licheniformis* 1001, *B. licheniformis* M017, *B. laterosporus*, and *B. amyloliquefaciens*, respectively. Post-data-processing, the Global Natural Product Social (GNPS) export files, i.e., GNPS MGF file and GNPS Sample Table (feature quantification table) were uploaded into the GNPS environment^2^ *via* the WinSCP server for molecular networking.

Feature-based molecular networks (FBMN) were generated using the respective workflow in the GNPS ecosystem. The set parameters used for generating different FBMN were precursor ion mass tolerance of 0.5 Da and fragment ion mass tolerance of 0.5 Da for *B. laterosporus* and *B. amyloliquefaciens B. licheniformis* M017, *B. licheniformis* 1001, and consortium. The cosine score was set to be above 0.6 and a minimum of 4 matched fragment ions. The resultant molecular networks were then enhanced with the MolNetEnhancer (Ernst et al., 2019) to improve the chemical structural annotations by incorporating *in silico* tools such as substructure recognition topic modeling (MS2LDA, MS2 latent Dirichlet allocation (Van Der Hooft et al., 2016) with MotifDB (Rogers et al., 2019) for annotated substructure patterns), DEREPLICATOR (Mohimani et al., 2018) and network annotation propagation (NAP) (da Silva et al., 2018). All the GNPS job links are provided in Supplementary Material. The networks were visualized using the Cytoscape network visualization tool/software (version 3.8.2) (Shannon et al., 2003; Smoot et al., 2011).

### Metabolite Annotation

Metabolite annotation was performed using semi-/automated and manual approaches. Automated metabolite annotation was carried out by searching the MS-DIAL metabolomics MSP spectral kits (All public MS/MS libraries)^3^ using MS-DIAL software. Further automated metabolite annotation was performed by searching against GNPS libraries *via* FBMN, DEREPLICATOR, and NAP which searched mass spectral and structural databases such as GNPS, HMDB, SUPNAT, CHEBI, and DRUGBANK. NAP jobs were run using the fusion scores and consensus score based on the first 10 candidates. MS2LDA interface in GNPS was used to explore and annotate substructures. Annotated MotifSets from MotifDB, such as MassBank, GNPS, *Euphorbia*, *Streptomyces* and *Salinisporus*, and *Photorhabdus* and *Xenorhabdus*, were included in the substructure discovery. The advanced MS2LDA parameters were set at default settings, overlap score threshold of 0.3, probability score of 0.1 and TopX of 5. Further details including which MotifDB annotated MotifSets were used for positive ionisation mode and the number of free motifs used can be found in the GNPS job links provided in the Supplementary Material.

To validate and improve the semi-/automated annotations, manual spectral annotation was performed using a multistep workflow consisting of (i) the calculation of molecular formula (MF) of a selected *m/z*, rt feature using Masslynx software (ii) searching of the MF candidates against databases and bioinformatics tools such Chemspider^4^ and KEGG,^5^ (iii) structural confirmation through careful inspection of fragmentation patterns by examining the MS^1^ and MS*^E^* spectra of the selected metabolite candidate and (iv) comparative assessment against annotation information of metabolites reported in the literature. Metabolites were annotated to levels 2 and 3 as classified by the Metabolomics Standard Initiative (MSI; (Sumner et al., 2007). All validated metabolites are listed in Supplementary Tables 1, 2.

## Results and Discussion

### Differentiation and Metabolome Annotation of *Bacillus* Strains

Decoding and knowing metabolite structures are pivotal to unraveling and understanding the functions of metabolites in (biological) systems (Beniddir et al., 2021). This study employed mass spectrometry, an indispensable MS fragmentation approach that results in MS/MS spectra, which could be regarded as metabolite structural fingerprints (Beniddir et al., 2021). The spectral data collected from all the selected time points (3, 7.5, 24, and 31.5 h growth incubation, Supplementary Figure 1) of *Bacillus* isolates and consortium were used to generate feature-based molecular networks (FBMNs)—the sizes of the resulting FBMNs varied across the different strains and consortium (Supplementary Figure 2). The most extensive network belonged to *B. laterosporus* (3898 spectral nodes, 496 spectral families, Supplementary Figure 2), followed by *B. amyloliquefaciens* (2,985 spectral nodes, 405 spectral families, Supplementary Figure 2), *B. licheniformis* M017 (602 spectral nodes, 55 spectral families, Supplementary Figure 2), *B. licheniformis* 1001 (566 spectral nodes, 48 spectral families, Supplementary Figure 2), and consortium (542 spectral nodes, 53 spectral families, Supplementary Figure 2). FBMN captured the structural diversity (represented as arranged mass spectra) existing within the chemical space of the four *Bacillus* isolates and the consortium (Supplementary Figure 2). The varying network sizes of the four *Bacillus* isolates and the consortium are suspected to be due to sample complexity and/or analytical variations.

The computed FBMNs also revealed putative annotations through an automated library spectral matching against the GNPS public spectral libraries, which found a match for 54 out of 3,898 spectral nodes of *B. laterosporus*, 127 out of 2,985 spectral nodes of *B. amyloliquefaciens*, 40 out of 602 spectral nodes of *B. licheniformis* M017, 26 out of 566 spectral nodes of *B. licheniformis* 1001, and 22 out of 542 spectral nodes of consortium (Supplementary Figure 2; GNPS job links provided in Supplementary Material). These library matches were scrutinized and the validated metabolite classes included amino acids and peptides, lipids, organic acids, nucleotides, vitamins and sugar (Supplementary Table 1). Although still very useful, FBMN in combination with spectral library matching enabled the annotation of only a small fraction of microbial MS/MS spectra, thus highlighting one of the major limitations in untargeted metabolomics. Furthermore, this demonstrates that current spectral libraries are still limited; and despite the development of computational tools, the vast majority of chemical signatures remain uncharacterized, and not all experimental spectra have a direct, exact match to the existing spectra libraries. However, even without fully identical spectral matches in the current libraries, the exploration of scaffolds or substructures (as inferred from the fragmentome) can still provide structural information of metabolites in a sample (Beniddir et al., 2021).

**FIGURE 1:**
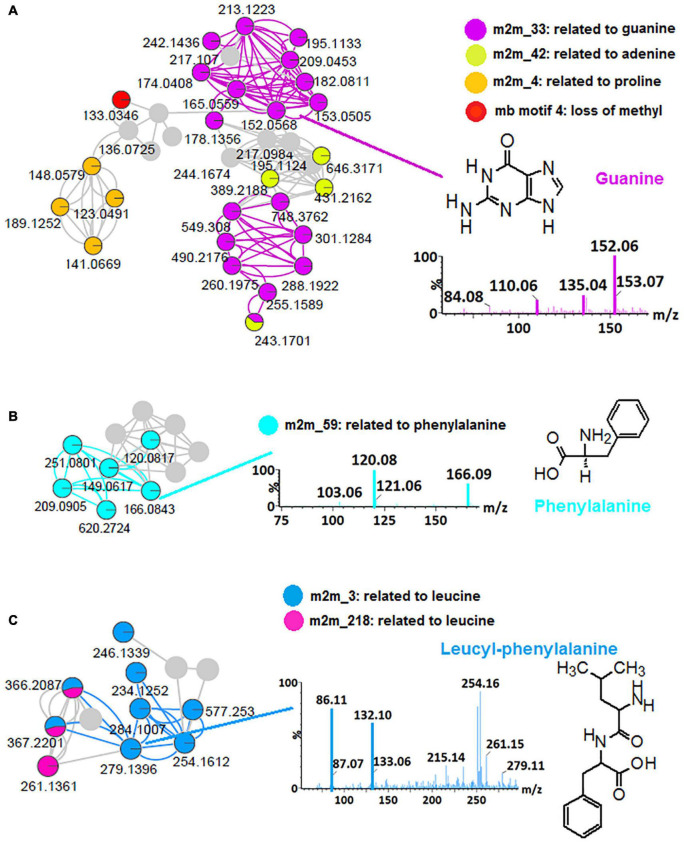
MS2LDA-driven metabolite annotation. Subnetworks extracted from an MS2LDA-enhanced molecular network of positive electrospray ionization (ESI^+^) MS/MS spectra obtained from *B. laterosporus* methanolic extracts (Supplementary Figure 3). Colored nodes represent the recognized substructures related to Mass2Motifs (m2m), **(A)** m2m_33: related to guanine, m2m_42: related to adenine, m2m_4: related to proline and mb motif4: loss of methyl group, **(B)** m2m_59: related to phenylalanine, **(C)** m2m_3 and m2m_218 related to leucine. The mass spectral network views were obtained *via* the MolNetEnhancer workflow.

Thus, to further explore the chemical space of the *Bacillus* (isolates and consortium), *in silico* annotation tools such as substructure recognition topic modeling (MS2LDA, MS2 Latent Dirichlet Allocation), and the NAP were performed for all spectral datasets from all four bacterial strains and a consortium, respectively. To illustrate the contribution of these tools to the microbial metabolite annotation and identification at a scaffold diversity level, the outputs obtained from *B. laterosporus* dataset are reported herein (Figure 1 and Supplementary Figure 3). MS2LDA is a machine learning method that performs an unsupervised decomposition of fragment spectra, revealing patterns of co-occurring fragments and neutral losses (termed “Mass2Motifs,” or shortly “m2m”) from multiple MS/MS spectra to extract information on substructural diversity within each class of metabolites (Van Der Hooft et al., 2016; Rogers et al., 2019). Five hundred and forty-one (541) Mass2Motif entries (chemical substructures) were learned and extracted from the *B. laterosporus* dataset with MS2LDA in GNPS—of which 168 were annotated and visualized *via* ms2lda.org (Wandy et al., 2018) into CytoScape (Supplementary Figure 3). Infographics shown in Figure 1 highlight some MotifDB-annotated motifs that were found in our LC-MS/MS data: m2m_42, related to adenine, m2m_4, related to proline, mb motif 4, related to loss of methyl group and m2m_33, which is indicative of guanine (Figure 1A), m2m_59, which is indicative of phenylalanine (Figure 1B) and m2m_3 and m2m_218 which are indicative of leucine (Figure 1C). The identification of motif m2m_3 helped with the annotation of the previously unidentified (unmatched *via* FBMN) features such as *m/z* 279, *m/z* 366 and *m/z* 577, which are within the molecular family cluster of leucine-related compounds (Figure 1C), *m/z* 279 was annotated as leucyl-phenylalanine (Figure 1C), *m/z* 366 as leucyl-phenylalanyl-serine and *m/z* 577 as glycine-glycine-serine-aspartic acid-leucine-glutamic acid, using the knowledge of m2m_3 from the MS2LDA. These annotations are recorded in Supplementary Table 1.

The NAP tool exploits the network topology to rearrange candidate structure lists based on neighboring matches within molecular families (Ernst et al., 2019). To obtain candidate structures, NAP uses *in silico* fragmentation performed with MetFrag, which searches for metabolites in structure databases such as GNPS, DNP (Dictionary of Natural Products), ChEBI (Chemical Entities of Biological Interest) and SUPER NATURAL II (Ernst et al., 2019). Then, NAP uses two scoring methods to re-rank the candidates: (i) fusion scoring—utilization of MetFrag *in silico* prediction with the MetFusion when there is a spectral library match within a molecular family of the molecular network and (ii) consensus scoring—which exploits the structural similarity from *in silico* candidates across the spectral nodes of a molecular family. Thus, NAP is also useful when there are no or very few spectral library matches, allowing the propagation of annotations even without the spectral matches to reference MS/MS data. In this study, we employed NAP onto the FBMN of *B.laterosporus* to search against the above-mentioned structure databases—using both the scoring methods. After manual validation using the fragmentation patterns and molecular formula prediction in MarkerLynx and literature, we found that NAP was able to come up with reliable annotations for nine (9) molecular entities (out of 1,258 features), i.e., metabolites such as tyrosine, phenylalanine, tryptophan, L-prolyl-L-isoleucine and guanosine,2’-deoxy (Supplementary Table 2).

**FIGURE 2:**
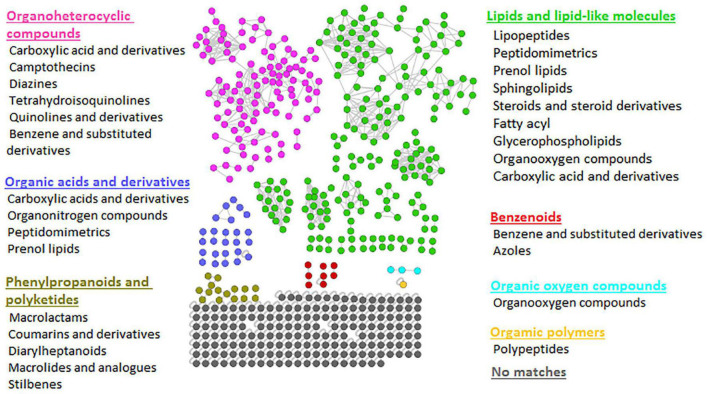
Representative MolNetEnhancer mass spectral feature-based network of consortium showing the chemical superclasses (written in colors corresponding to the nodes) and classes (written in black) that were putatively annotated based GNPS library matches and enhanced with substructure annotations (MS2LDA), network annotation propagation (NAP), and DEREPLICATOR outputs. The MolNetEnhancer networks of *Bacillus* isolates are shown in Supplementary Figures 4, 5.

The FBMN workflow was further integrated with *in silico* annotation tools such as MS2LDA, NAP, and DEREPLICATOR, generating enhanced (MolNetEnhancer) molecular networks. The latter reveals molecular families, subfamilies, and subtle structural differences between family members (Ernst et al., 2019), thus allowing increased confidence in the class annotation and level of biochemical interpretation. MolNetEnhancer networks provided more comprehensive chemical insights into each *Bacillus* strain’s measured metabolomes and the consortium, respectively (Figure 2 and Supplementary Figures 4, 5).

To explore the chemical diversity of the *Bacillus* metabolome, we looked at the class ontology level, which revealed various metabolite classes to be present, as shown in Figure 2. For example, when looking at lipid and lipid-like molecules, metabolite classes such as lipopeptides, peptidomimetrics, prenol lipids, sphingolipids, glycerolipids, etc. were shown in the consortium (Figure 2). However, we note that some of these metabolite classes are hardly recorded before in *Bacillus* species. For instance, there are limited studies reporting on sphingolipids in *Bacillus* species. Currently, there is only one existing study reporting the presence of sphingolipids (sphinganine and phytosphingosine) in *Bacillus* spp.—sphinganine and phytosphingosine were identified using Accurate-Mass Q-TOF LC-MS, the detected masses and retrieved chemical formulae were further compared with the standard compounds in the Metlin database (Gao et al., 2016). The latter reported that *B. cereus* exhibited high nematicidal activity against *Meloidogyne incognita* by producing sphingosine (Gao et al., 2016). Moreover, the sphingolipid metabolism pathway in *B. subtilis* exists in KEGG database^6^; however, these pathways lack the identification of enzymes that are directly responsible for the synthesis of sphingolipids in *B. subtilis*. Nonetheless, such findings suggest the possibility of sphingolipids being synthesized by *Bacillus* species. As is true for all *in silico* approaches, further confirmatory studies are required to validate the existence of metabolite superclasses and classes annotated by the MolNetEnhancer strategy (Figure 2 and Supplementary Figures 4, 5). Thus, our findings provide, for the first time, a global metabolic landscape that describes the chemotypes of agriculturally important *Bacillus* strains. Furthermore, evaluating the chemical space of different *Bacillus* strains, i.e., their metabolic potentials in regards to the biosynthesis of certain metabolites (e.g., lipopeptides and sphingosines) can innovatively contribute toward the design of a novel combination of *Bacillus* strains for a biostimulant consortium formulation. For instance, with the primer knowledge generated from our study regarding the possibility of the biosynthesis of sphingolipids in *Bacillus* species, more studies will build on such insights to further establish mechanistically the sphingolipids biosynthesis pathways in *Bacillus* spp. Such actionable knowledge is a necessary step toward the design of novel specific biostimulant formulations containing the sphingolipids-producing *Bacillus*, which would exhibit maximum nematocidal activity.

### Lipid and Lipid-Like Molecules—Lipopeptides: A Bioactive Molecular Family of Interest in *Bacillus-*Plant Interaction

**FIGURE 3:**
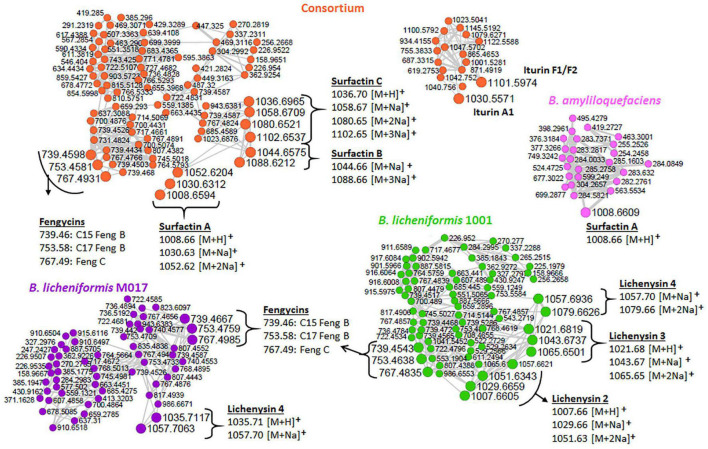
Lipopeptide clusters extracted from the networks of isolates and the consortium (full networks in Supplementary Figure 2). Different node colors represent different strains, orange (consortium), pink (*B. amyloliquefaciens*), green (*B. licheniformis* 1001), and purple (*B. licheniformis* M017). Feng, fengycin.

**FIGURE 4:**
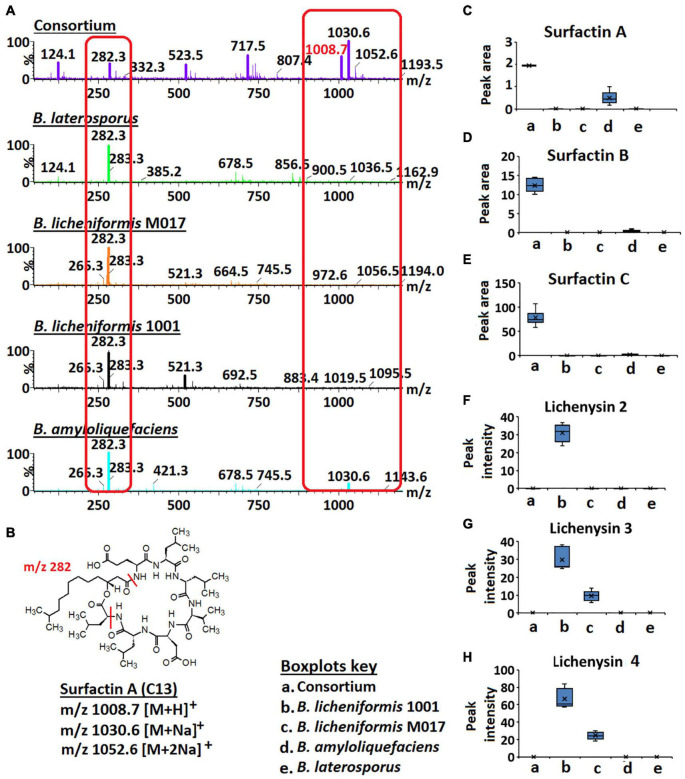
Full MS scans of *m/z* 1008.7/1030.6 (surfactin A) and relative quantification of surfactins and lichenysins. **(A)** Full MS scans of *m/z* 1008.7/1030.6 (surfactin A) in *Bacillus* consortium and *Bacillus* isolates, *B. amyloliquefaciens*, *B. licheniformis* 1001, *B. licheniformis* M017, and *B. laterosporus*. Increased ion intensity of *m/z* 1008.7/1030.6 is observed in the consortium whereas its fragment (m/z 282.3) is decreased (highlighted in red boxes). **(B)** Structure of surfactin A. **(C–H)** Boxplots showing the relative quantification of surfactins A–C and lichenysins 2, 3, and 4 in the *Bacillus* strains and the consortium.

As revealed by the generated MolNetEnhancer networks of the *Bacillus* isolates and the consortium (Figure 2 and Supplementary Figures 4, 5), the lipid and lipid-like molecules were the most predominant superclass (e.g., 25 out of 53 spectral families in the consortium (Figure 2). Within this superclass, the lipopeptide class can be found (Figure 2)—these latter are cyclic compounds known to be present in *Bacillus* strains, including members of the surfactin, lichenysin, iturin, and fengycin families (Bernat et al., 2016). Interestingly, we observed several lipopeptide molecular families with differential abundance between the various strains and consortium. The surfactin family cluster was more apparent in the consortium (Figure 3) whereas, the lichenysin family cluster was more apparent in the *B. licheniformis* strains (Figure 3). The consortium revealed three surfactins with mass-to-charge ratio (*m/z*) 1008.7 [M + H]^+^, *m/z* 1044.7 [M + Na]^+^ and *m/z* 1058.7 [M + Na]^+^, which were manually annotated [based on fragmentation fingerprints and available literature (Nguyen et al., 2013), see methodology] as surfactin A, surfactin B, and surfactin C, respectively (Figure 3). The *B. licheniformis* 1001 network revealed three (3) lichenysins, with *m/z* 1007.7 [M + H]^+^, *m/z* 1021.7 [M + H]^+^ and *m/z* 1057.7 [M + Na]^+^, which were manually annotated (based on fragmentation fingerprints and available literature, see methodology) as lichenysin 2, lichenysin 3, and lichenysin 4, respectively (Figure 3). The *B. licheniformis* M017 network showed lichenysin 4 only (Figure 3). Surfactins and lichenysins were not detected at all in *B. laterosporus* strain (Figure 4).

Further investigation of surfactins across isolates and consortium showed that the signal at *m/z* 1008.7/1030.6 (annotated as ion species of surfactin A—Figure 3) had a higher ion intensity in the consortium vs. in isolates (Figures 4A,C). Inversely, the signal at *m/z* 282.3 showed a higher ion intensity in the isolates compared to the consortium (Figure 4). *m/z* 282.3 was determined to be a lipopeptidic fragment of surfactin A (Figure 4B). Moreover, the increased levels of surfactin B (*m/z* 1022.7/1044.7) and surfactin C (*m/z* 1036.66/1058.66) was also observed in the consortium vs. in isolates (Figures 4D,E). Such observations suggest that the combination/co-culture of *Bacillus* strains encourages increased biosynthesis of surfactins. Coincidingly, similar results were found in the study by Zhi et al. (2017), where co-culturing of two *B. amyloliquefaciens* strains, MT45 and X82 yielded 50% increase of surfactin production. Surfactins can function as signaling molecules in the intraspecific intercommunication of *Bacillus* cells—these lipopeptides can interact with the cell membranes—and their presence influences biofilm formation (López et al., 2009; Zhi et al., 2017; Chen et al., 2020). Interestingly, a study by Zhi et al. (2017) showed that biofilm formation is negatively correlated to increased surfactin synthesis, indicating a complex interplay between molecular factors. Moreover, the authors speculated that biofilm formation interferes with the interaction between pheromone ComX and histidine kinase ComP, thereby directly suppressing the transcription of surfactin synthetase gene (Zhi et al., 2017). Supported by the findings of Zhi et al. (2017), our results suggest that the selected *Bacillus* strains (*B. licheniformis* M017, *B. licheniformis* 1001, *B. amyloliquefaciens*, and *B. laterosporus*) are perfect co-culture partners to promote surfactin synthesis that in turn can promote the formation of protective biofilms and can also hamper the growth of pathogenic microbes by penetrating their membranes. Thus, such information (i.e., microbial intraspecific interactions) can be harnessed and utilized to develop commercially valuable formulations for the agricultural, bioremediation, cosmetics, or pharmaceutical industries.

In contrast to surfactins, lichenysin 2, 3, and 4 were only found in the monocultures of *B. licheniformis* 1001 and *B. licheniformis* M017, respectively, and were not reflected in the consortium (Figures 4F–H). These results suggest that co-culturing of *B. licheniformis* M017, *B. licheniformis* 1001, *B. amyloliquefaciens*, and *B. laterosporus* does not support the synthesis of lichenysins (Figures 4F–H). Moreover, lichenysin 2, 3, and 4 were found in higher levels in *B. licheniformis* 1001 than *B. licheniformis* M017, suggesting that *B. licheniformis* 1001 is a better lichenysins producer (Figures 4F–H).

The network clusters in which surfactins and lichenysins (in consortium and *B. licheniformis* strains, respectively) were annotated showed nodes representing the *m/z* 739.45, 753.45, and 767.47 (Figure 3), suggesting a spectral similarity between the mentioned spectral nodes and surfactin and lichenysin family. Thus, this information was used to further explore the identity of these three nodes (*m/z* 739.45, *m/z* 753.45, and *m/z* 767.47). The study of Nasfi et al. (2018) revealed that these three nodes are doubly charged ion species that represent C15 fengycin B2 (*m/z* 1477.83), C17 fengycin B (*m/z* 1505.85), and fengycin C, respectively. The putative annotation of fengycins nicely illustrates that FBMN allows the annotation of unknown spectral nodes through spectral similarity. Furthermore, our findings suggest that the other unannotated nodes within these clusters (Figure 3) are structurally related to lipopeptides, and such information can help assign names to more unannotated nodes. Moreover, another family of lipopeptides that was putatively annotated in the metabolome of *Bacillus* strains is the iturin family—notably, correctly subclassified as dipepsipeptides by MolNetEnhancer (distinct from the surfactin, lichenysin and fengycin cluster) (Figure 2). The application of MolNetEnhancer aided in assigning names to two nodes (within the iturin dipepsipeptides cluster), annotating *m/z* 1101.6 and *m/z* 1030.5 annotated as iturin F1/F2 and iturin A1, respectively, in all isolates and the consortium networks (Figure 3). Overall, with the assistance of molecular networking tools, we could annotate several lipopeptides from *Bacillus* isolates and the consortium: three variants of surfactins (surfactin A, surfactin B, and surfactin C), three variants of lichenycins (lichenycin 2, lichenycin 3 and lichenycin 4), three variants of fengycins (fengycin B2, fengycin B, and fengycin C) and two variants of iturins (iturin F1/F2 and iturin A1) (Figure 3).

The pursuit of discovering and exploring *Bacillus* lipopeptides has long been an attractive undertaking for scientists and industry researchers due to its various applications such as interference with flagella development, affecting the bacterial adhesion, inhibition of biofilm formation and disruption of pre-formed biofilms (Hu et al., 2019; Englerová et al., 2021). Moreover, the bioactivity of lipopeptides comes from the capability of these cyclic compounds to disrupt the structures and functions of bio-membranes, which improves membrane permeability—one of the key factors important in *Bacillus*-plant chemical intercommunication (Bernat et al., 2016). For example, surfactins represent a class of lipopeptides that have been extensively studied. A recent study by Andrić et al. (2021) demonstrated that *Bacillus* mobilizes its surfactin to improve motility and reduce the toxicity of *Pseudomonas* by acting as chemical deactivators of *Pseudomonas* lipopeptides, sessilins and tolaasins.

Moreover, *Bacillu*s surfactins have also been shown to promote a symbiotic relationship with other beneficial bacterial species, thus shaping the plant microbiome. For example, the study by Luzzatto-Knaan et al. (2019), demonstrated the role of surfactins as interspecies recruitment factor—where surfactins recruited *Paenibacillus dendritiformis* to its ecological niche. In this study, the authors applied imaging mass spectrometry (IMS) and molecular networking to elucidate the exact molecular mechanisms involved in the chemotaxis of *P. dendritiformis* toward *B. subtilis*. One of the exact mechanisms demonstrated was that, *P. dendritiformis* actively breaks down *B. subtilis*-produced surfactins. Moreover, the degradation products (lipopeptidic fragment *m/z* 636.42 [M + Na]^+^ and peptidic fragment *m/z* 459.28 [M + H]^+^) of surfactin C were demonstrated to serve as territorial markers and also indicated the metabolic exchange between the interacting partners (Luzzatto-Knaan et al., 2019). In other words, characterizing the intracellular metabolome of the bacteria (PGPR in this case), provides insights necessary for the biostimulant industry. In the same philosophy, interrogating the metabolome of the biostimulant consortium (reported in our study) indicated differential metabolic profiles compared to *Bacillus* isolates/individual strains. Such inisghts pave a way to possibilities of designing and formulating different *Bacillus* combinations, novel microbial biostimulants.

### Differential Metabolic Charts of *Bacillus* Strains at Different Growth Stages and Longitudinal Lipopeptide Profiles

The timing of production and the distribution of metabolites within microbial populations can provide valuable insight into the function of specific molecules (Watrous et al., 2012). Thus, in this study, *Bacillus* strains were cultured in an LB media (experimental section) and the growth progression was monitored over time (Supplementary Figure 1). The cells were harvested at different time points, corresponding to a bacterial growth stage (Supplementary Figure 1). The 3-h (3 h) extract represents the lag phase (Supplementary Figure 1), described as a “delayed” growth stage whereby the cells are preparing to adapt and exploit new environmental conditions. This growth stage is assumed to include the process of restoring damaged macromolecules and the biosynthesis of cellular components necessary for cell differentiation and growth (Rolfe et al., 2012). The 7.5 h extract represents the log phase also known as the exponential phase (Supplementary Figure 1)—a stage in which the microbial cells are rapidly dividing, thus being more energy-demanding (Rolfe et al., 2012; Aliashkevich et al., 2018). Lastly, the 24 and 31.5 h extracts represent the stationary phase (Supplementary Figure 1)—a bacterial growth stage whereby cells are both actively dividing as well as dying (Raad et al., 2021).

**FIGURE 5:**
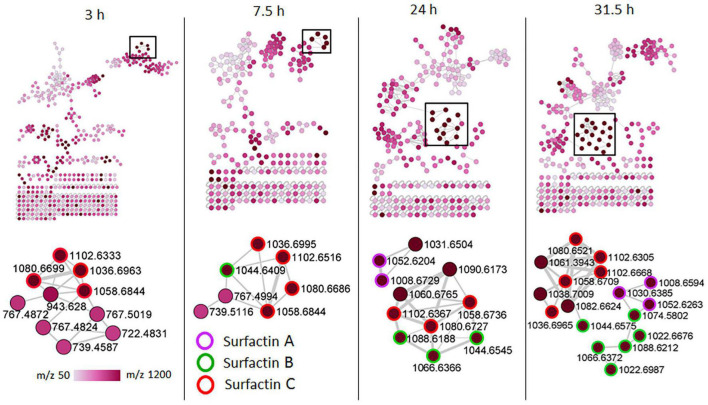
Molecular networking of positive electrospray ionization (ESI^+^) MS/MS spectra obtained from consortium over time. The zoom-in snaps show that the presence of lipopeptides (surfactins) varies depending on the bacterial growth phase. The nodes are colored based on the acquired mass range (*m/z* 50–1,200 Da) of the precursor ions: light pink nodes represent the smallest masses whereas the dark purple nodes represent the largest masses.

**FIGURE 6:**
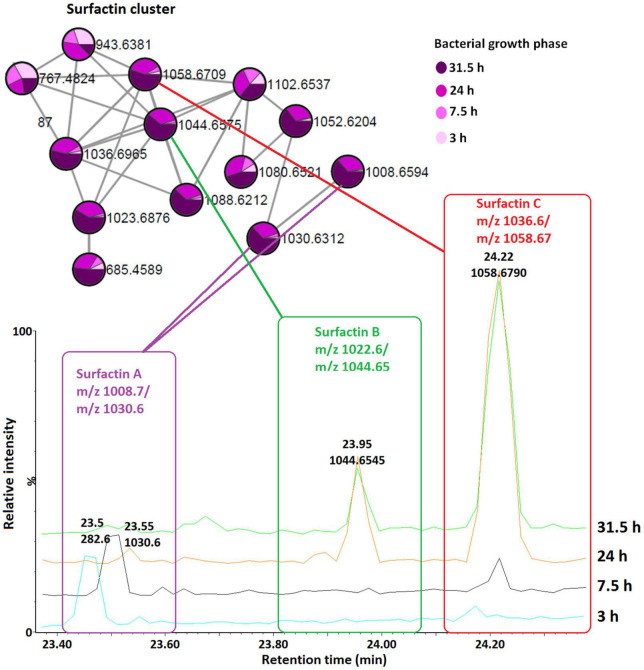
Qualitative and relative quantification of surfactins in the consortium over time (bacterial growth phases). Surfactin A, B, and C are present in large amounts in the late growth stages (24 and 31.5 h, stationary phase) compared to the early growth stages (lag, 3 h and log, 7.5 h).

To examine the temporal changes of the metabolic features in *Bacillus* strains, FBMN was applied (Figure 5 and Supplementary Figures 7–9). The generated MNs showed differential network topology at each time point, reflecting a reprogramming of the bacterial metabolism at various growth stages (Figure 5 and Supplementary Figures 7–9). As shown above (Figure 4), the surfactins are predominantly present in the consortium, followed by *B. amyloliquefaciens* (Figure 4), thus time-dependent FBMN was applied to qualitatively evaluate the presence of surfactins at different growth stages of cells in the consortium and *B. amyloliquefaciens* (Figure 5 and Supplementary Figure 7). Time-dependent FBMNs clearly showed that the surfactin variant with a longer lipid chain (Surfactin C, *m/z* 1036.69/1058.67) was present in the consortium (Figures 5, 6) and *B. amyloliquefaciens* (Supplementary Figure 7) across the whole-time course, from the lag- to stationary phase (Supplementary Figure 1). Moreover, surfactin C is the most abundant surfactin variant in the consortium, as depicted by the peak area (Figures 4C–E, 6). The surfactin with the second-longest lipid chain, surfactin B (*m/z* 1022.68/1044.66), was also present throughout the bacterial growth time course (3, 7.5, 24, and 31.5 h) in *B. amyloliquefaciens* (Supplementary Figure 7), whereas in the consortium, surfactin B was present at log phase (7.5 h) and stationary phase (24 and 31.5 h) only (Figure 5). Corresponding findings were obtained in the study by Watrous et al. (2012), where surfactin variants with longer lipid chains were found throughout the growth stages of *B. subtilis*.

The surfactin with a shorter lipid chain (surfactin A, *m/z* 1008.6/1030.6), in contrast, only appeared in the consortium at the stationary phase, at 24 and 31.5 h (Figures 5, 6), whereas in *B. amyloliquefaciens*, surfactin A appeared after 7.5 h incubation (log phase) and remained present at the stationary phase (24 and 31.5 h) (Supplementary Figure 7). The relative quantification evaluation of surfactins in the consortium showed that all surfactin variants, surfactin A, B, and C levels are higher at the stationary phase (24 and 31.5 h) compared to the lag (3 h) and log phase (7.5 h) (Figure 6 and Supplementary Figure 6).

*B. licheniformis* 1001 and *B. licheniformis* M017 were characterized by lichenysin variants and surfactins were not observed (Figure 3). However, upon performing time-dependent FBMN, surfactins were found in *B. licheniformis* 1001 and *B. licheniformis* M017 (Supplementary Figures 8, 9). All three surfactin variants, surfactin A, B, and C were detected in *B. licheniformis* 1001 at the stationary phase (24 h) (Supplementary Figure 8), whereas in *B. licheniformis* M017, only surfactin C was present at the lag phase (3 h) (Supplementary Figure 9). Lichenysin 2, 3, and 4 (*m/z* 1007.7, *m/z* 1021.7/1043.7, *m/z* 1035.7/1057.7, respectively) were present in *B. licheniformis* 1001 at the log (7.5 h) and stationary phase (24 and 31.5 h) (Supplementary Figure 8), whereas no lichenysins nor surfactins were found at the lag phase (3 h) in *B. licheniformis* 1001 (Supplementary Figure 8). *B. licheniformis* M017 showed only lichenysin 2, 3, and 4 at the stationary phase (24 h) (Supplementary Figure 9). Moreover, *B. licheniformis M017* showed only lichenysin 4 at the log phase (7.5 h) and only lichenysin 2 at the stationary phase (31.5 h) (Supplementary Figure 9). Similar to the surfactins in the consortium (Figure 5), lichenysin variant of a shorter lipid chain (lichenysin 2, m/z 1007.6/1029.6) was observed in *B. licheniformis* M017 at the late bacterial growth stages, stationary phase (24 and 31.5 h) (Supplementary Figure 9). These results show that time-dependent FBMN provides unique access into molecular information about microbial systems, such as the production of different lipopeptide variants in *Bacillus*.

Thus, the application of mass spectrometry and molecular networking tools allowed the decoding of *Bacillus* intracellular chemical space, revealing a metabolic landscape characterized by various chemical classes. Furthermore, investigating the chemical classification of the lipopeptide clusters (within the lipids and lipid-like molecules), led to the putative annotation of four (4) lipopeptides classes: lichenysins, surfactins, iturins, and fengycins. Based on the results of this study, it appears that surfactins are present in larger quantities in the consortium compared to the other annotated lipopeptides such as lichenysins iturins and fengycins (Figure 4). Furthermore, surfactins were present in high levels in the consortium compared to the isolates (Figures 4C–E). Surfactins have been proven to be involved in recruiting other organisms to their ecological niche (Luzzatto-Knaan et al., 2019) —such metabolic interplay suggests that plant inoculation with the consortium may be advantageous to establish a favorable mixed microbial community, thus, reinforcing synergistic collaborations in the plant microbiota.

Further interrogation of the lipopeptide clusters revealed that lichenysins were unique to *B. licheniformis* strains. Moreover, performing time-dependent MN revealed that surfactins are also present in *B. licheniformis* strains (Supplementary Figures 7, 8), which were not shown in the network with combined time points (Figure 3). Time-dependent MN also showed an increasing number of lipopeptides in all the isolates and the consortium over time, and the stationary phase (24 and 31.5 h) was identified as the bacterial growth phase whereby lipopeptides are present in large amounts in the cells (Figure 5 and Supplementary Figures 7–9). Such information can help lipopeptide production companies to achieve a commercial scale by reducing the high production and time costs, as those are identified as the main obstacles to the large-scale industrial application (Zhi et al., 2017). Furthermore, time course studies (Supplementary Figures 6–9), can help determine the overturn of functional metabolites in the bacterial formulations as well as the shelf-life of the products, thereby contributing to the development and commercialization of effective microbial formulations (Aamir et al., 2020).

We do recognize that our study includes several limitations: (i) since untargeted metabolomics approach was applied to perform a wide-screen study, relative quantification was performed; and a targeted metabolomics study is recommended for validation and absolute quantification of selected subset of metabolites (e.g., lipopeptides); moreover, other complementary omics approaches such as gene expression could be integrated to gain further insights in molecular mechanisms; (ii) metabolite feature recognition and metabolites annotated *via* automated library matching still require substantial manual expert intervention; (iii) some of the chemical classes identified through MolNetEnhancer still require further confirmation; (iv) microbial databases are still in the developmental phase [we recognize increasing efforts to collect and share curated microbial data, i.e., such as in the NP Atlas database, (Van Santen et al., 2019)], and submission of high-quality *Bacillus* annotated spectra to existing libraries (such as those present in GNPS) is crucial to obtaining the fully annotated map of the *Bacillus* metabolism—the latter could translate into a significant knowledge contribution to the science community and to commercial sectors, i.e., companies formulating *Bacillus*-based biostimulants; (v) the functions of annotated metabolites (e.g., lipopeptides) still require further investigation under real-world conditions (i.e., plant-*Bacillus* interactions). Some studies support that indeed plant-associated-*Bacillus* strains secrete the annotated lipopeptides (surfactins, iturins, and fengycins) to the rhizosphere. For example, the study by Nihorimbere et al. (2012) demonstrated that lipopeptides secreted by root-adhering *Bacillus* cells bind with high affinity to plant cell membrane and remain tightly associated with the membrane structure of plant cells. Another study by Chowdhury et al. (2015) demonstrated that surfactins and other lipopeptides produced by *B. amyloliquefaciens* were secreted to the lettuce rhizosphere and were proven to be involved in the disease suppression by regulating the plant defense genes toward Bottom Rot pathogen, *Rhizoctonia solani*. Despite these limitations, our study has provided unique insights into the microbial chemical space of plant-associated bacteria that will pave the way for future studies that further confirm and assess our current findings. Future studies could include (i) identifying the exact mechanisms (i.e., impacted pathways) involved in enhancing surfactin biosynthesis in the consortium due to synergistic interaction of isolates and (ii) investigating the correlation between the enhanced surfactin biosynthesis and the surfactin secretion to the extracellular space. Such studies will contribute toward understanding the chemical intercommunication between the PGPR-secreted surfactins and the plant.

The lack of high-throughput annotation tools has been one of the constraining factors in untargeted metabolomics, limiting the chemotyping of agricultural plant growth-promoting rhizobacteria (PGPR) and mechanistic understanding of the belowground microbe-plant interactions. In this study, the application of metabolome mining tools found in the GNPS environment enabled putative annotation and classification of metabolites in *Bacillus* strains and consortium. Further interrogation of lipopeptides revealed that co-culturing of *Bacillus* strains (*B. licheniformis* M017, *B. licheniformis* 1001, *B. amyloliquefaciens*, and *B. laterosporus*) are suitable co-culture partners to promote surfactin synthesis. Deeper understanding of the bacterial metabolome and metabolic pathways (i.e., surfactin biosynthesis pathway) that are activated during the interaction of isolates in the consortium can contribute to the elucidation of the exact plant growth-promoting properties. Moreover, the molecular networking approaches allowed the visualization of the metabolic snapshots of the bacteria at different time points, facilitating the characterization of the bacterial growth stages, which informatively and descriptively points out intracellular molecular circuits and processes occurring in the bacterial strains under study. This fundamental and actionable knowledge—metabolic profiles of *Bacillus* strains and consortium—contributes to understanding the chemical space of PGPR, which can inform on the bacterial strain resistance/persistence and also predict the performance of the formulated product under field conditions Ultimately, such efforts would help in designing the effective *Bacillus*-based formulations, eliminating chances of inconsistence and inadequate performance (Aamir et al., 2020). Moreover, our acquired knowledge of the bacterial chemical space and the described methodology can be used in various economic sectors such as the cosmetic, pharmaceutical, and food industries, apart from agricultural applications.

## Data Availability Statement

The datasets presented in this study can be found in online repositories. The names of the repository/repositories and accession number(s) can be found below: The study LC-MS data, GNPS MGF file, GNPS Feature Quantification Table, and metadata can be found on https://massive.ucsd.edu/, MSV000089107.

## Author Contributions

FT, KB, and JH conceived to the project and funding acquisition. KB, MB, and FT guided and co-ordinated the research. LN, PS, MB, KB, JJJvdH, and FT performed the experimental work, analysis, and interpretation of the data. LN and FT contributed to writing—original draft preparation. KB, JJJvdH, and FT contributed to writing—review and editing. All authors have read and agreed to the published version of the manuscript.

## Conflict of Interest

JJJvdH was member of the Scientific Advisory Board of NAICONS Srl., Milano, Italy. JH, MB, and FT work for Omnia Group Ltd., SA, which provided microbial isolates and consortium formulations. The remaining authors declare that the research was conducted in the absence of any commercial or financial relationships that could be construed as a potential conflict of interest.

## Publisher’s Note

All claims expressed in this article are solely those of the authors and do not necessarily represent those of their affiliated organizations, or those of the publisher, the editors and the reviewers. Any product that may be evaluated in this article, or claim that may be made by its manufacturer, is not guaranteed or endorsed by the publisher.

## Footnotes

1
https://www.reifycs.com/AbfConverter/

2
https://gnps.ucsd.edu/

3
http://prime.psc.riken.jp/compms/msdial/main.html#MSP

4
www.chemspider.com

5
www.genome.jp/kegg/
