# Supplementary material for: Mass Spectral Molecular Networking to Profile the Metabolome of Biostimulant Bacillus Strains
Source: Front Plant Sci. 2022 Jun 9;13:920963. doi: 10.3389/fpls.2022.920963 (PMC9218640; doi:10.3389/fpls.2022.920963)
Supplement: Supplementary file 1 [file Data_Sheet_1.PDF]

Supplementary Material

GNPS JOB LINKS

Feature- based molecular networking (FBMN) results

B. laterosporus
FBMN: https://gnps.ucsd.edu/ProteoSAFe/status.jsp?task=f3adf5ebe235432c8545fea546d4c392
MS2LDA: https://gnps.ucsd.edu/ProteoSAFe/status.jsp?task=f7ba9da7a5d04263bdcff52ebfd910cb
NAP: https://gnps.ucsd.edu/ProteoSAFe/status.jsp?task=546fdf8a502e4d3c8e8ade9ebb428f7b
Dereplicator: https://gnps.ucsd.edu/ProteoSAFe/status.jsp?task=0da473e3a7824f8fa8064118f7201840
MolnetEnhancer:
https://gnps.ucsd.edu/ProteoSAFe/status.jsp?task=b88d028e288f4428bc34714106ccd684

B. amyloliquefaciens
FBMN: https://gnps.ucsd.edu/ProteoSAFe/status.jsp?task=e0578f26d2914b48b43b2afb6bcf287f
MS2LDA: https://gnps.ucsd.edu/ProteoSAFe/status.jsp?task=f06bd0dff7bf417b889e2b5974101862
NAP: https://gnps.ucsd.edu/ProteoSAFe/status.jsp?task=edf1cac5feaa4aa8a4f45ee18508839b
Dereplicator: https://gnps.ucsd.edu/ProteoSAFe/status.jsp?task=e5cb8fa61703418292abc72f469bfb6a
MolnetEnhancer: https://gnps.ucsd.edu/ProteoSAFe/status.jsp?task=6bf6557e696e448eb6e1f619ddff7c59

B. licheniformis 1001
FBMN: https://gnps.ucsd.edu/ProteoSAFe/status.jsp?task=d1e2f9d292124bf1bb2ab6aeb8a9f37f
MS2LDA: https://gnps.ucsd.edu/ProteoSAFe/status.jsp?task=33de128e664e45fc9d33284b038a425d
NAP: https://gnps.ucsd.edu/ProteoSAFe/status.jsp?task=8ddc7f19b1f54591bbcb5e5f4d104016
Dereplicator: https://gnps.ucsd.edu/ProteoSAFe/status.jsp?task=81bcf23c966642d199d2edb616c16997
MolnetEnhancer:
https://gnps.ucsd.edu/ProteoSAFe/status.jsp?task=96af675f9d354f4d863bf7182b88e12b

B. licheniformis M017
FBMN: https://gnps.ucsd.edu/ProteoSAFe/status.jsp?task=9f0a83e90ad24527b00ea2b074a94a0c
MS2LDA: https://gnps.ucsd.edu/ProteoSAFe/status.jsp?task=ac56ada736a84e95b4900bdb308bb2ae
NAP: https://gnps.ucsd.edu/ProteoSAFe/status.jsp?task=64a04b12e93b4e038b44d90adfa1cf25
Dereplicator: https://gnps.ucsd.edu/ProteoSAFe/status.jsp?task=e348cedb15234e4abdb37d303c262541
MolnetEnhancer: https://gnps.ucsd.edu/ProteoSAFe/status.jsp?task=1fcba85fd77240858ffee89ed081d115

Consortium
FBMN: https://gnps.ucsd.edu/ProteoSAFe/status.jsp?task=1d5cef417c854783a6d98e435559cb84
MS2LDA: https://gnps.ucsd.edu/ProteoSAFe/status.jsp?task=d65f5a518cde4e2783f247e32011295b
NAP: https://gnps.ucsd.edu/ProteoSAFe/status.jsp?task=be948a2eb1924479bbf5df16a322b276
Dereplicator: https://gnps.ucsd.edu/ProteoSAFe/status.jsp?task=7bec1cf6a66b42e9b1dc09ab718cc5d2
MolnetEnhancer:
https://gnps.ucsd.edu/ProteoSAFe/status.jsp?task=508fd4c080754961b702369ebc9f862a

Time-specific FBMN results

B. laterosporus 3h:
https://gnps.ucsd.edu/ProteoSAFe/status.jsp?task=7d905692e1aa49b38b43d26e14d3575f
B. laterosporus 7.5h:
https://gnps.ucsd.edu/ProteoSAFe/status.jsp?task=aa539c793d5343cd905b0b40ad6e0095
B. laterosporus 24h:
https://gnps.ucsd.edu/ProteoSAFe/status.jsp?task=4986826a43a34e9e89657aa987925e73
B. laterosporus 31.5h:
https://gnps.ucsd.edu/ProteoSAFe/status.jsp?task=06206f9cc2f04564ace9d49036dec455

B.licheniformis M017 3h:
https://gnps.ucsd.edu/ProteoSAFe/status.jsp?task=81005f96098549469027fd3410f99302
B.licheniformis M017 7.5h:
https://gnps.ucsd.edu/ProteoSAFe/status.jsp?task=ca830ff66a084bb1aa8906a61d6dd89e
B.licheniformis M017 24h:
https://gnps.ucsd.edu/ProteoSAFe/status.jsp?task=92ab86ed5dbc4313a536c334c5e99f70
B.licheniformis M017 31.5h:
https://gnps.ucsd.edu/ProteoSAFe/status.jsp?task=85690e9ac8a64b3288f2a18996b9d0c2

B. licheniformis 1001 3h:
https://gnps.ucsd.edu/ProteoSAFe/status.jsp?task=247c424b6ed84853b57e1aef76ea6cb8
B. licheniformis 1001 7.5h:
https://gnps.ucsd.edu/ProteoSAFe/status.jsp?task=bd92377f001144248b27c19f994049ae
B. licheniformis 1001 24h:
https://gnps.ucsd.edu/ProteoSAFe/status.jsp?task=107b109fec664e1baedaba34fe13e3d1
B. licheniformis 1001 31.5h:
https://gnps.ucsd.edu/ProteoSAFe/status.jsp?task=8f94a7d023e14596af83f5c01e7c5958

Consortium 3h:
https://gnps.ucsd.edu/ProteoSAFe/status.jsp?task=35c8246b6e3248b7bd0936a1f9a31682
Consortium 7.5h:
https://gnps.ucsd.edu/ProteoSAFe/status.jsp?task=a29921a5242441dd90f5ce507a99f73b
Consortium 24h:
https://gnps.ucsd.edu/ProteoSAFe/status.jsp?task=9ea8346d2a554a078609c82638346cd0
Consortium 31.5h:
https://gnps.ucsd.edu/ProteoSAFe/status.jsp?task=c37600da332a4e799f8ac09dbd5f8f3c

B. amyloliquefaciens 3h
https://gnps.ucsd.edu/ProteoSAFe/status.jsp?task=b58cf483a19740aab6e40b31628e9863
B. amyloliquefaciens 7.5h:
https://gnps.ucsd.edu/ProteoSAFe/status.jsp?task=b58cf483a19740aab6e40b31628e9863
B. amyloliquefaciens 24h:
https://gnps.ucsd.edu/ProteoSAFe/status.jsp?task=04933f32bd274adb911f19e8c6fab02e
B. amyloliquefaciens 31.5h:
https://gnps.ucsd.edu/ProteoSAFe/status.jsp?task=fe630d4276934ae5b1966e8c93fe920c

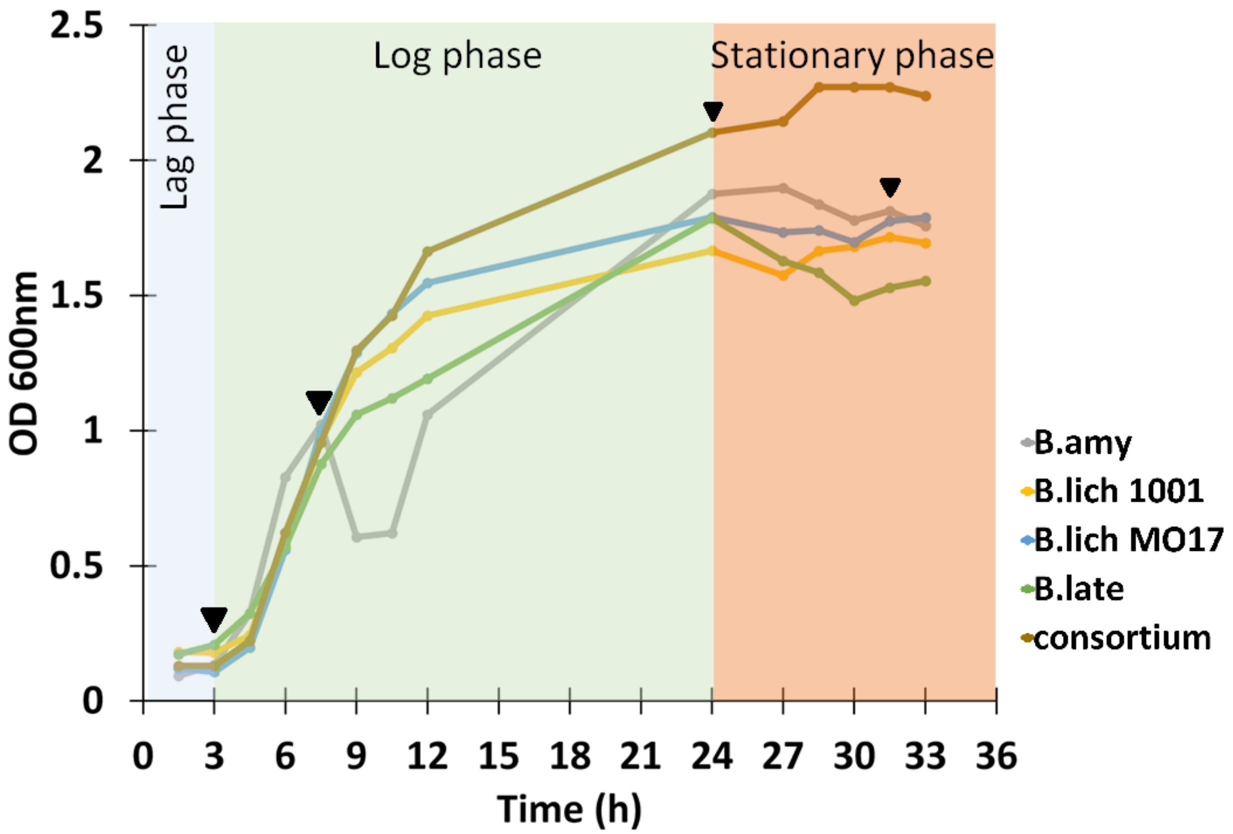

Figure S1. Growth rates of *B. laterosporus* (B. late), *B. amyloliquefaciens* (B.amy), *B. licheniformis* 1001 (B. lich 1001), *B. licheniformis* M017 (B. lich M017) and consortium. Arrows represent harvesting time points. The spectral data from all the selected time points (3, 7.5, 24 and 31.5 h growth incubation) of *Bacillus* isolates and consortium were used to generate feature-based molecular networks (FBMNs).

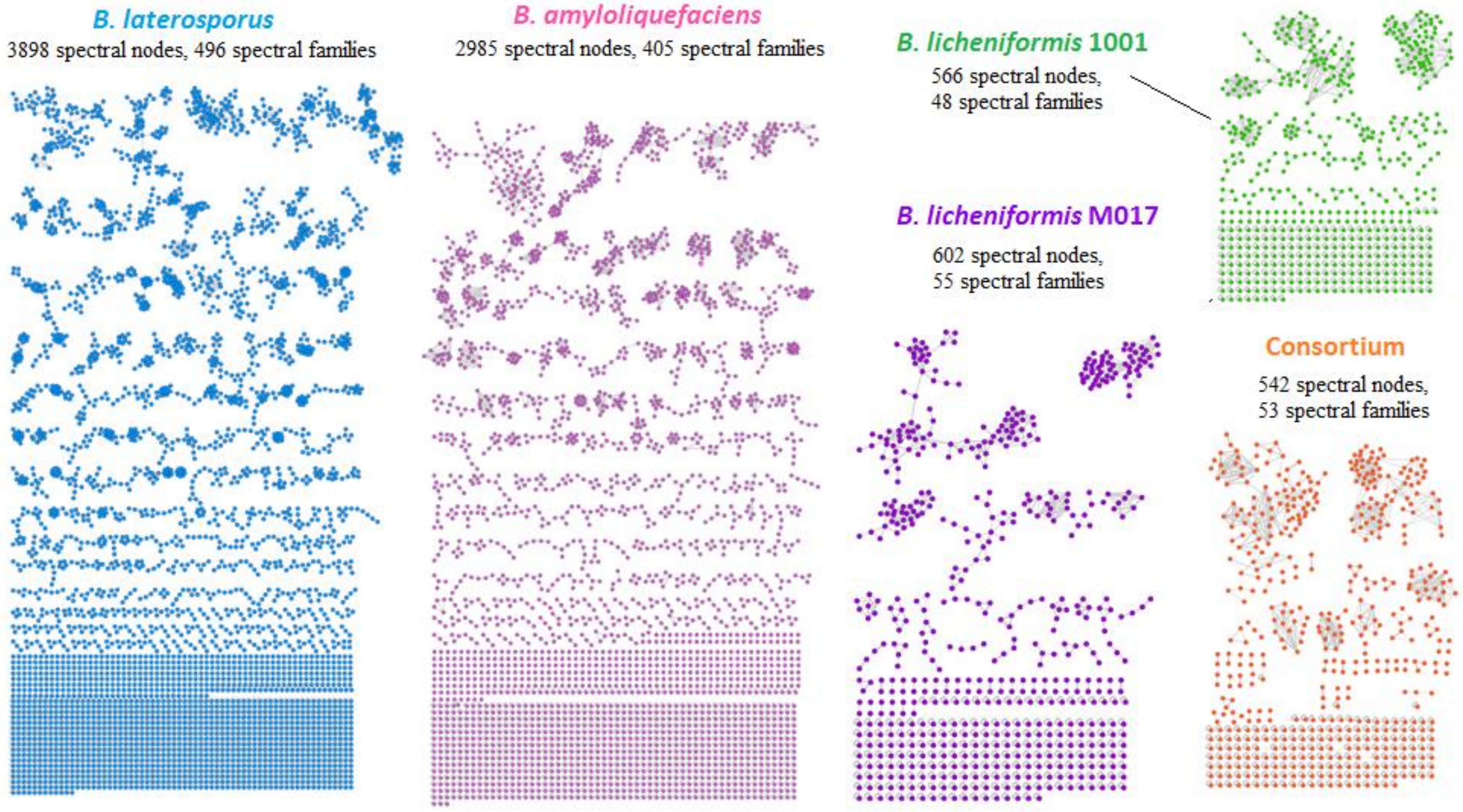

Figure S2. Feature-based molecular networks of positive electrospray ionisation (ESI$^+$) MS/MS spectra obtained from *B. amyloliquefaciens*, *B. laterosporus*, *B. licheniformis*1001, and *B. licheniformis* M017 strains, and the consortium.

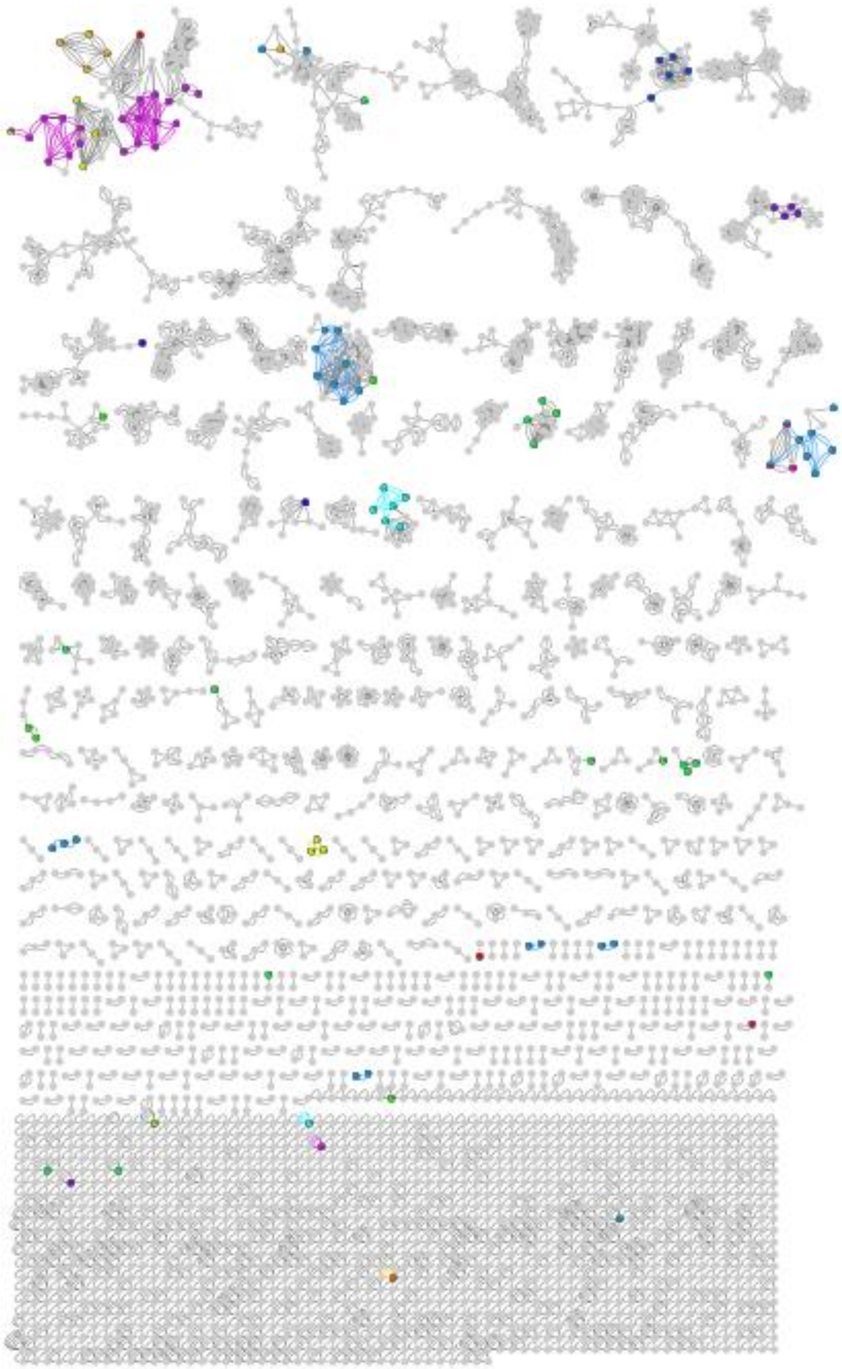

Figure S3. MS2LDA network of positive electrospray ionisation (ESI^{+}) MS/MS spectra obtained from *B. laterosporus* methanolic extract.

Table S1: Metabolite annotation table. Colour coding: FBMN-GNPS library matches are written in **red**, MS2LDA-driven metabolite annotations are written in **green,** **a**nnotations from DEREPLICATOR written in **blue** and manually annotated metabolites are written in '**black**'.

No.	Compound name	Formula	rt (min)	m/z	Fragments	Adduct/ ion mode
**Amino acids and peptides**						
**1**	Glycine betaine	$C_5H_{11}NO_2$	0.83	118.0883	84,59	$[M+H]^+$
**2**	Proline	$C_5H_9NO_2$	0.86	116.07	70	$[M+H]^+$
**3**	Alanylvaline	$C_8H_{16}N_2O_3$	0.96	189.12	118,112,84,70	$[M+H]^+$
**4**	Tyrosine	$C_9H_{11}NO_4$	1.52	182.059	166,165,147,136,123,120,91	$[M+H]^+$
**5**	Leucine	$C_6H_{13}NO_2$	1.72	132.13	86	$[M+H]^+$
**6**	Phenylalanine	$C_9H_{11}NO_2$	2.091	166.09	120,103	$[M+H]^+$
**7**	Leucylproline	$C_{11}H_{20}N_2O_3$	2.81	229.1581	132	$[M+H]^+$
**8**	Isoleucyl-proline-isoleucine	$C_{17}H_{31}N_3O_4$		342.24	324,229,211,183,130,86	$[M+H]^+$
**9**	Tryptophan	$C_{11}H_{12}N_2O_3$	3.49	205.036	189,188,170,169,146,144,132,118	$[M+H]^+$
**10**	Cyclo(L-Tyr-L-Pro)	$C_{14}H_{16}N_2O_3$		261.13	233,155,136	$[M+H]^+$
**11**	Cyclo(Leu-Phe)	$C_{15}H_{20}N_2O_2$	5.29	261.12	-	$[M+H]^+$
**12**	Cyclo(leucylprolyl)	$C_{11}H_{18}N_2O_2$		211.14	183,130,86,70	$[M+H]^+$
**13**	Leucyl-phenylalanyl-serine	$C_{18}H_{27}N_3O_5$	1.75	366.21	261, 251,195,166,132,86	$[M+H]^+$
**14**	Leucyl-phenylalanine	$C_{15}H_{22}N_2O_3$	1.81	279. 13	261,254,251,132,86	$[M+H]^+$
**15**	Gly-Gly-Ser-Asp-Leu-Glu	$C_{22}H_{36}N_6O_{12}$	1.69	577.05	288,146,132,86	$[M+H]^+$
**16**	6-Aminocaproic acid	$C_6H_{13}NO_2$		113.97	96,72,69	$[M+H-H_2O]^+$
**17**	p-Hydroxyphenyllactic acid	$C_9H_{10}O_4$		165.06	148,147,137,136,123,119,95,91	$[M+H-H_2O]^+$
**Antimicrobials**						
**18**	Basiliskamide A	$C_{23}H_{31}NO_4$	8.94	386.24	216,216,149	$[M+H]^+$
**19**	7-O-Succinyl macrolactin A	$C_{28}H_{38}O_8$	9.1	525.25	385,367,349	$[M+Na]^+$
**20**	Bacillaene B	$C_{34}H_{48}N_2O_6$	9.33	605.29	604,578,489,447	$[M+Na]^+$

**21**	Oxydifficidin	$C_{31}H_{45}O_7P$	10.09	561.27	560,545	$[M+H]^+$
**22**	Macrolactin U	$C_{31}H_{44}O_4$	10.34	503.27	243,230,217	$[M+Na]^+$
**23**	Iturin A	$C_{48}H_{74}N_{12}O_{14}$	15.57	1043.83	827,653,583,245	$[M+H]^+$
**24**	Hetiamacin B	$C_{23}H_{33}N_3O_7$	17.89	486.2	463	$[M+Na]^+$
**25**	Macrolactin A	$C_{24}H_{35}O_5$	18.31	425.22	385,367,349	$[M+Na]^+$
**26**	7-O-Malonyl macrolactin A	$C_{27}H_{36}O_8$	18.53	511.27	425,385,367,349	$[M+Na]^+$
**27**	Bacilysocin	$C_{21}H_{43}O_9P$	20.85	471.27	453,435,332,299	$[M+H]^+$
**28**	Gageotetrin B	$C_{38}H_{70}N_4O_9$	22.271	728.46/727.46	639,597,595,507,464,313,299	$[M+H]^+$
**29**	Surfactin A (C13)	$C_{51}H_{89}N_7O_{13}$	23.5	1030.66	1008.6,767,745,711,686,543,523	$[M+H]^+$
**30**	Surfactin B (C14)	$C_{52}H_{91}N_7O_{13}$	23.96	1044.66	1022,1021,839,717,685,551,523,304	$[M+Na]^+$
**31**	Surfactin C (C15)	$C_{53}H_{93}N_7O_{13}$	24.22	1058.67	1036,1018,685	$[M+Na]^+$
**32**	Surfactin C15 dimethyl ester	-		1046.67	947,937,903,838,820,790,721,677,483,469	$[M+H]^+$
**32**	Lichenysin 2		23.75	1007.66	745,590,332,304,284,142,124,102	$[M+H]^+$
**33**	Lichenysin 3		24.2	1043.67	1021, 745,590,332,304,284,142,124,102	$[M+Na]^+$
**34**	Lichenysin 4	$C_{52}H_{91}N_8O_{12}$	24.47	1057.69	1034,607,507,339,330,304,142,124,102	$[M+Na]^+$
**35**	Iturin F1/F2		13.04	1101.597		
**36**	Iturin A1		13.161	1030.557		
**37**	C15 Feng B		23.556	739.4598		
**38**	Feng C		23.637	767.4931		
**39**	C17 Feng B		23.785	753.4581		
**Hormones and derivatives**						
**40**	Indoleacrylic acid	$C_{11}H_9NO_2$	3.51	188.07	146,118	$[M+H]^+$
**41**	(2S)indoline-2-carboxylic acid	$C_9H_9NO_2$		146.06	118	$[M+H-H_2O]^+$
**42**	Indole-3-Acetamide	$C_{10}H_{10}N_2O$		197.13	-	$[M+Na]^+$
**Lipids**						
**43**	12-HOME	$C_{18}H_{34}O_3$	20.89	299.25	195,137	$[M+H]^+$

**44**	Oleamide	$C_{18}H_{35}NO$		282.28	266,265,184,156,	$[M+H]^+$
**45**	Oleic acid	$C_{18}H_{34}O_2$		265.25	247,163,149,135,121,107,95,81,69	$[M+H-H_2O]^+$
**46**	Vaccenic acid	$C_{18}H_{34}O_2$		283.28	282	$[M+H]^+$
**47**	1-tridecanoyl-2-hydroxy-sn-glycero-3-phosphocholine	$C_{21}H_{44}NO_7P$		454.29	436,104	$[M+H]^+$
**48**	Eicosapentaenoic acid	$C_{20}H_{30}O_2$		303.25	285	$[M+H]^+$
**49**	Palmitamide	$C_{16}H_{33}NO$		256.263	102,88	$[M+H]^+$
**50**	Lipoamide	$C_8H_{15}NOS_2$		228.23	-	$[M+Na]^+$
**Nucleotides**						
**51**	Guanine i	$C_5H_5N_5O$	0.93	152.06	136,135,110	$[M+H]^+$
**52**	Xanthine	$C_5H_4N_4O_2$	1.58	153.04	136,135,110	$[M+H]^+$
**53**	8-Hydroxy-7-methylguanine	$C_6H_7N_5O_2$	0.94	182.09	166.165,152,188	$[M+H]^+$
**54**	Deoxyguanosine	$C_{10}H_{13}N_5O_4$	1.65	268.1	226,209136,135,110	$[M+H]^+$
**55**	Guanine ii	$C_5H_5N_5O$	1.69	152.06	136,135,110	$[M+H]^+$
**56**	Adenine	$C_5H_5N_5$		136.08	119,94	$[M+H]^+$
**57**	Methyladenosine	$C_{11}H_{15}N_5O_4$		282.28	265	[M+H]+
**Organic acids**						
**58**	Oxalosuccinate	$C_6H_6O_7$	3.39	228.9736	191	$[M+K]^+$
**59**	Citric acid	$C_6H_8O_7$		215.02		$[M+Na]^+$
**Other compounds**						
**60**	2-(2-oxo-8,9-dihydrofuro[2,3-h]chromen-8-yl)propan-2-yl acetate	$C_{16}H_{16}O_5$		306.134	304	$[M+NH_4]^+$
**61**	[(2E,4E)-4,6-Dimethyl-2,4-octadienoyl]-2,3-dihydroxy-2-methylcyclohexanone	-		317.27	299	$[M+Na]^+$
**62**	4-methoxy-9-(3-methylbut-2-enyl)furo[3,2-g]chromen-7-one	$C_{17}H_{16}O_4$		302.139	-	$[M+NH_4]^+$

**63**	Nicotinate/vitamin B3	$C_6H_4NO_2^-$	124.04	123,96,80	$[M+H]^+$
**64**	Alpha-Tocopherol	$C_{29}H_{50}O_2$	453.34		$[M+Na]^+$
**65**	Biotin	$C_{10}H_{16}N_2O_3S$	262.12	261	$[M+NH_4]^+$
**66**	4-Hydroxyquinoline	C_9H_7NO	146.06	118	$[M+H]^+$
**67**	4-hydroxy-2-heptylquinoline	$C_{16}H_{21}NO$	244.13	-	$[M+H]^+$
**68**	4-Hydroxy-5,7-dimethyl-3-benzylhydroquinolin-2-one	-	302.19	-	$[M+Na]^+$
**69**	E)-8-(4-hydroxy-6-methoxy-7-methyl-3-oxo-1H-2-benzofuran-5-yl)-2,6-dimethyloct-6-enoic acid	$C_{20}H_{26}O_6$	385.24	229	$[M+Na]^+$
**70**	Fructose	$C_6H_{12}O_6$	228.00	129	$[M+Na]^+$

Table S2: NAP results for *B. laterosporus*. Manually validated metabolites are indicated in green.

Cluster.index	m/z	RT	LibraryID	MetFragSC	FusionSC	ConsensusSC
**43**	118.087	0.844	BETAINE	Lipids and lipid-like molecules#Organic acids and derivatives#Organic nitrogen compounds	Organoheterocyclic compounds	Organic acids and derivatives
**40**	116.071	0.886	L-PROLINE	Organic acids and derivatives	Organic acids and derivatives	Organic acids and derivatives
**98**	165.059	1.572	SpectralMatchtop-HydroxyphenyllacticacidfromNIST14	Lipids and lipid-like molecules#Organic acids and derivatives#Organosulfur compounds	Lipids and lipid-like molecules#Organic acids and derivatives#Organosulfur compounds	Lipids and lipid-like molecules#Organic acids and derivatives#Organosulfur compounds
**97**	182.079	1.586	SpectralMatchtoL-TyrosinefromNIST14	Organic acids and derivatives#Organoheterocyclic compounds#Organic oxygen compounds	Organic compounds#Organic acids and derivatives	Organic compounds#Organic acids and derivatives
**62**	136.074	1.587	Massbank:LU0852011H-Benzotriazol-1-ol|1-hydroxybenzotriazole	Benzenoids		Benzenoids#Organic oxygen compounds
**117**	182.083	1.587	SpectralMatchtoL-TyrosinefromNIST14	Organic acids and derivatives#Organoheterocyclic compounds#Organic oxygen compounds	Organic compounds#Organic acids and derivatives	Organic compounds#Organic acids and derivatives
**181**	268.104	1.649	SpectralMatchtoGuanosine,2'-deoxy-fromNIST14	Organic compounds#Benzenoids#Phenylpropanoids and polyketides#Organoheterocyclic compounds		Organic compounds#Benzenoids#Phenylpropanoids and polyketides#Organoheterocyclic compounds
**26**	120.079	2.088	MassbankEU:SM836003Indoline|Indoline|2,3-dihydro-1H-indole			
**46**	239.168	2.091	SpectralMatchtoBenzoicacid,3-amino-fromNIST14			
**99**	166.083	2.093	Massbank:PR311156Phenylalanine			
**188**	231.171	3.05	MassbankEU:SM855001Propyphenazone|1,5-dimethyl-2-phenyl-4-propan-2-ylpyrazol-3-one	Organic acids and derivatives	Organic acids and derivatives	Organic compounds

**144**	205.096	3.497	D-TRYPTOPHAN-20.0eV	Organoheterocyclic compounds#Organic acids and derivatives		Organic acids and derivatives#Organic compounds#Organoheterocyclic compounds
**124**	188.071	3.526	SpectralMatchtoDL-Indole-3-lacticacidfromNIST14	Benzenoids#Organic acids and derivatives#Organoheterocyclic compounds#Organic compounds	Organoheterocyclic compounds	Organoheterocyclic compounds#Benzenoids
**177**	229.153	11.14	L-Prolyl-L-isoleucine	Organic acids and derivatives#Organic compounds	Organic acids and derivatives#Organic compounds	Organic acids and derivatives#Organic compounds
**176**	229.151	13.595	L-Prolyl-L-isoleucine			
**322**	354.155	15.988	Massbank:NA002377Protopin|Protopine|15-methyl-7,9,19,21-tetraoxa-15-azapentacyclo[15.7.0.04,12.06,10.018,22]tetracosa-1(17),4,6(10),11,18(22),23-hexaen-3-one	Organoheterocyclic compounds#Benzenoids	Organoheterocyclic compounds#Benzenoids	Benzenoids#Alkaloids and derivatives#Organoheterocyclic compounds
**1177**	855.5	17.045	Massbank:LQB00685PI36:5	Phenylpropanoids and polyketides	Phenylpropanoids and polyketides	Phenylpropanoids and polyketides
**899**	716.483	17.807	SpectralMatchto1-Hexadecanoyl-2-(9Z-octadecenoyl)-sn-glycero-3-phosphoethanolaminefromNIST14			
**1270**	883.525	18.093	Massbank:LQB00317PI38:5	Organoheterocyclic compounds	Organoheterocyclic compounds	Organoheterocyclic compounds
**293**	339.152	18.661	OlopatadineHCl	Organoheterocyclic compounds#Benzenoids	Organoheterocyclic compounds#Benzenoids	Organic acids and derivatives#Organic compounds
**241**	299.255	20.163	methyloctadecanoate	Lipids and lipid-like molecules#Organosulfur compounds	Lipids and lipid-like molecules#Organic acids and derivatives	Lipids and lipid-like molecules#Organic acids and derivatives
**240**	299.253	20.409	methyloctadecanoate			
**461**	454.291	20.411	Massbank:RP0261011-palmitoyl-2-hydroxy-sn-glycero-3-phosphoethanolamine|1-palmitoyl-sn-glycero-3-phosphoethanolamine|2-	Organic compounds		Benzenoids

			azaniumylethyl[(2R)-3-hexadecanoyloxy-2-hydroxypropyl]phosphate			
**242**	299.256	20.875	methyloctadecanoate	Lipids and lipid-like molecules	Lipids and lipid-like molecules#Organic acids and derivatives	Lipids and lipid-like molecules#Organic acids and derivatives
**169**	282.278	23.497	Oleamide	Lipids and lipid-like molecules#Organic acids and derivatives	Lipids and lipid-like molecules#Organic acids and derivatives	Lipids and lipid-like molecules#Organic acids and derivatives

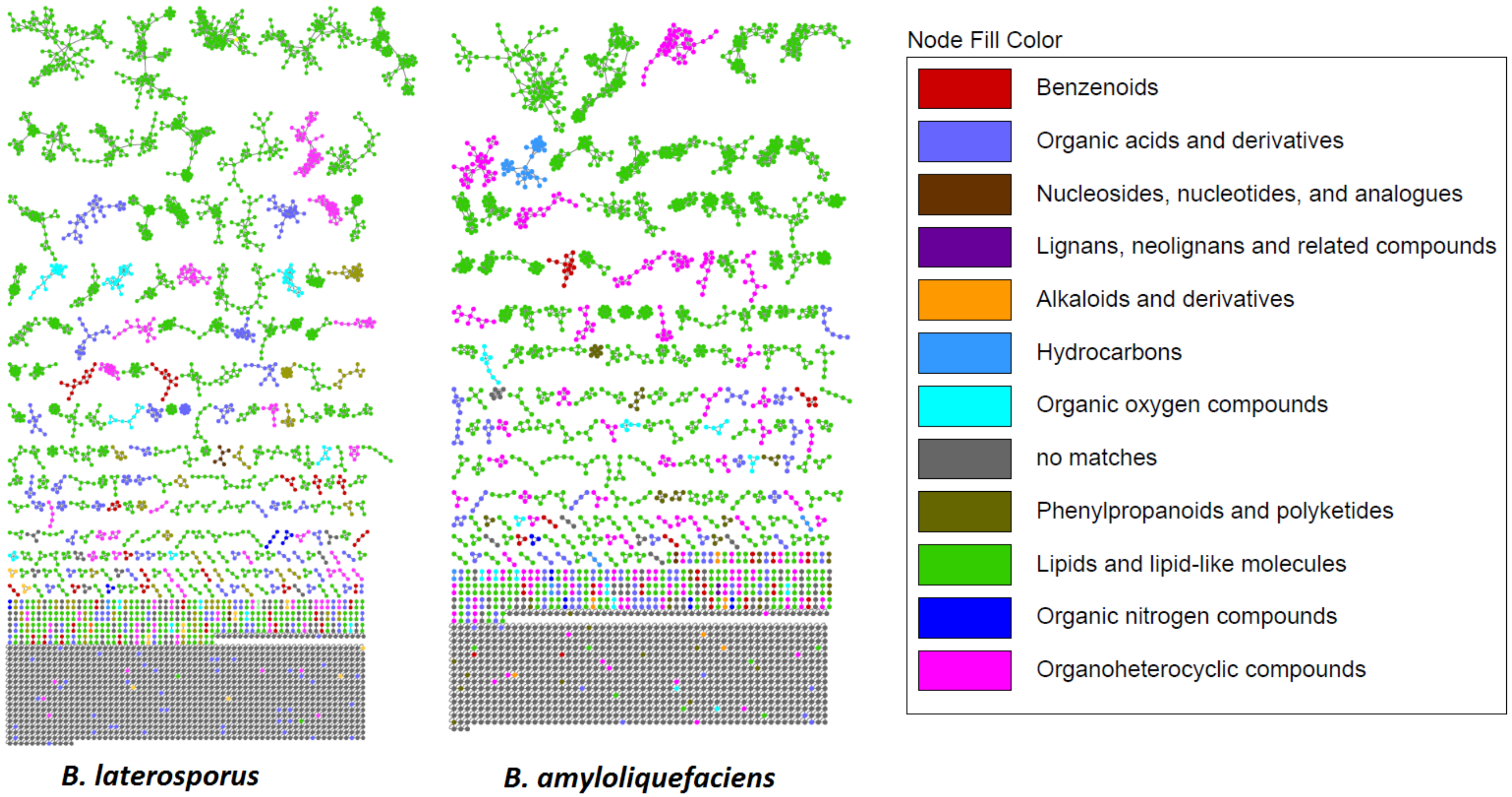

Figure S4. MolnetEnhancer of positive electrospray ionisation (ESI$^+$) MS/MS spectra obtained from *B. amyloliquefaciens* and *B. laterosporus* showing the chemical superclasses that were putatively annotated based GNPS library matches and enhanced with substructure annotations (MS2LDA), network annotation propagation (NAP) and DEREPLICATOR outputs.

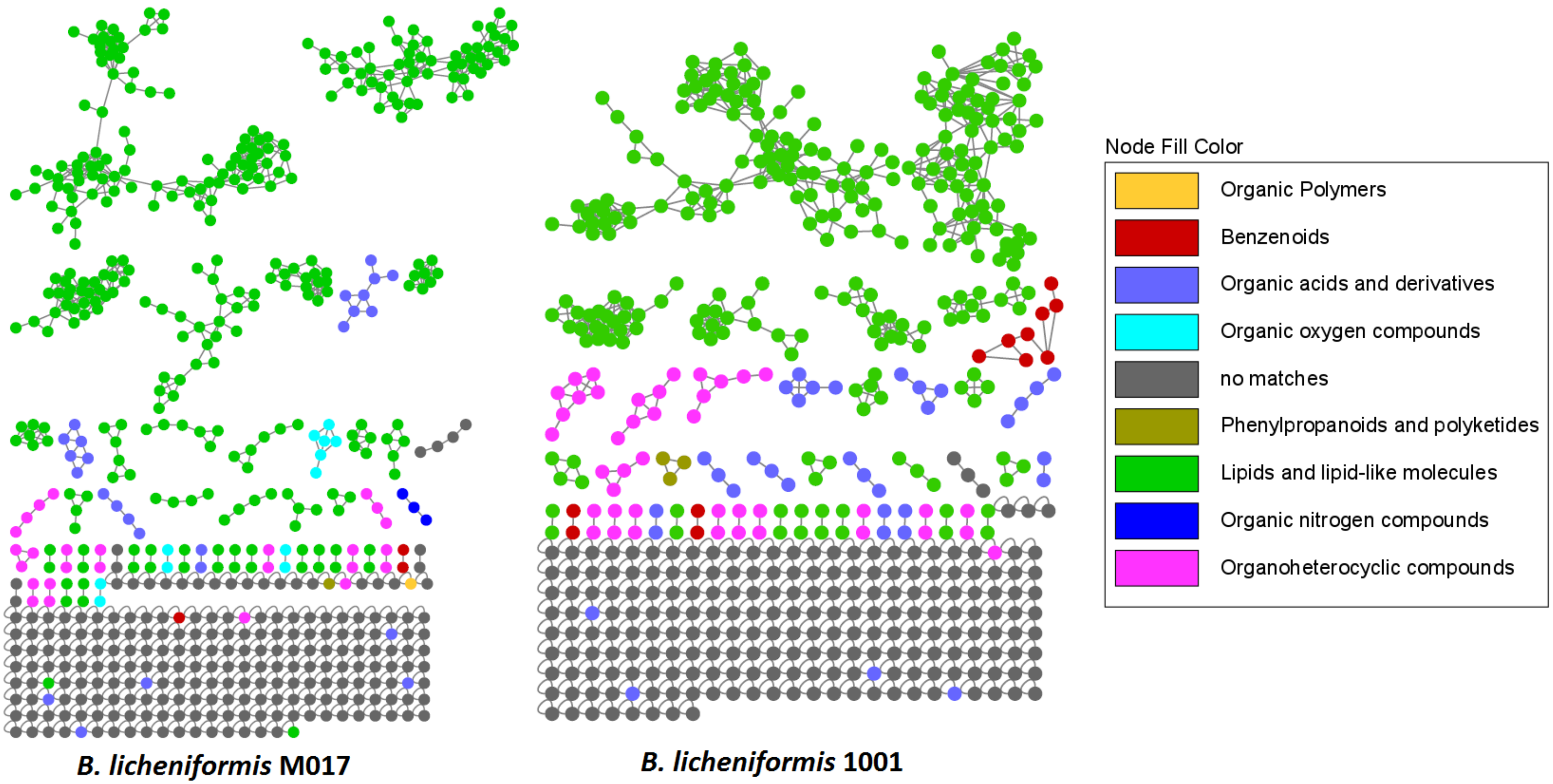

Figure S5. MolnetEnhancer of positive electrospray ionisation (ESI+) MS/MS spectra obtained from *B.licheniformis*1001 and *B.licheniformis* M017 showing the chemical superclasses that were putatively annotated based GNPS library matches and enhanced with substructure annotations (MS2LDA), network annotation propagation (NAP) and DEREPLICATOR outputs.

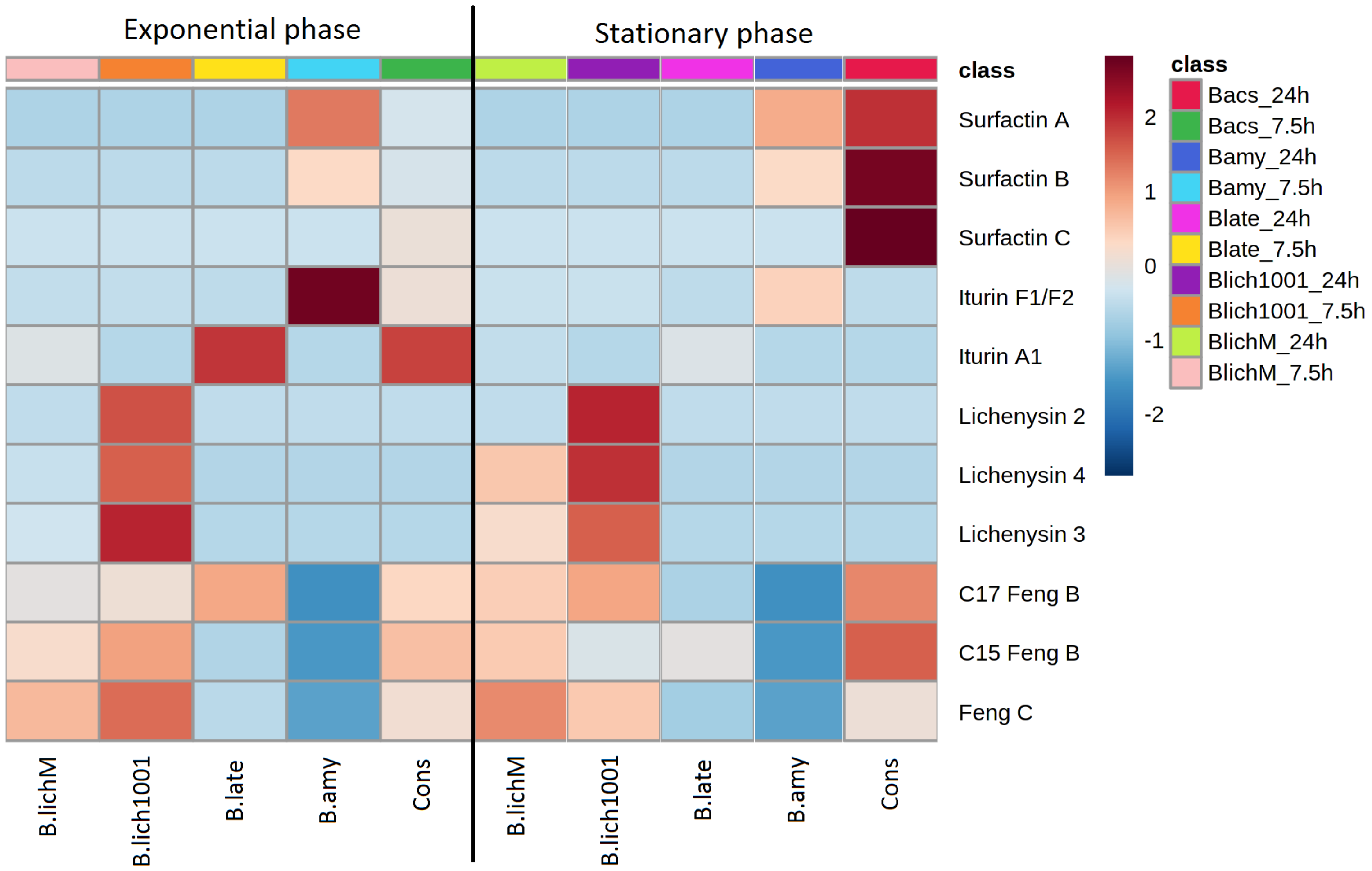

Figure S6. A heatmap showing the relative abundance of lipopeptides annotated from the intracellular milieu of *Bacillus* isolates and consortium. B.lichM = *B. licheniformis* M017, B.lich1001 = *B. licheniformis* 1001, B. late = *B. laterosporus*, B.amy = *B. amyloliquefaciens*, Cons = consortium, exponential phase (7.5 h) and stationary phase (24 h).

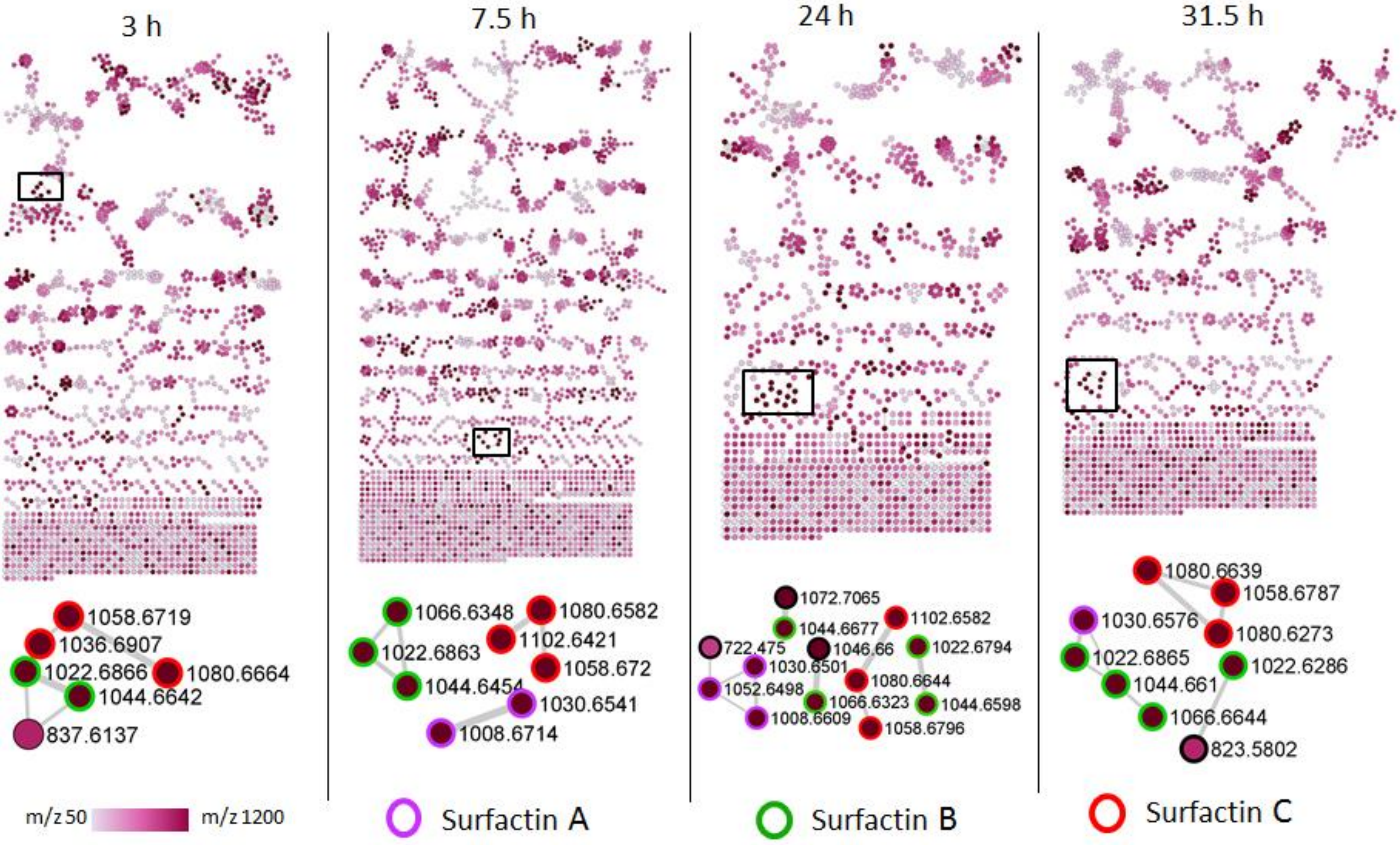

Figure S7. **Molecular networking of positive electrospray ionisation (ESI$^+$) MS/MS spectra obtained from *B. amyloliquefaciens* over time.** The zoom-in snaps shows that presence and type of lipopeptides varies depending on the bacterial growth phase. The stationary phase (24 h) showed more lichenysins. The nodes are coloured based on the acquired mass range (*m/z* 50-1200 Da) of the precursor ions: light pink nodes represent the smallest masses whereas the dark purple nodes represent the largest masses. Surf = surfactin and Lich = lichenysin.

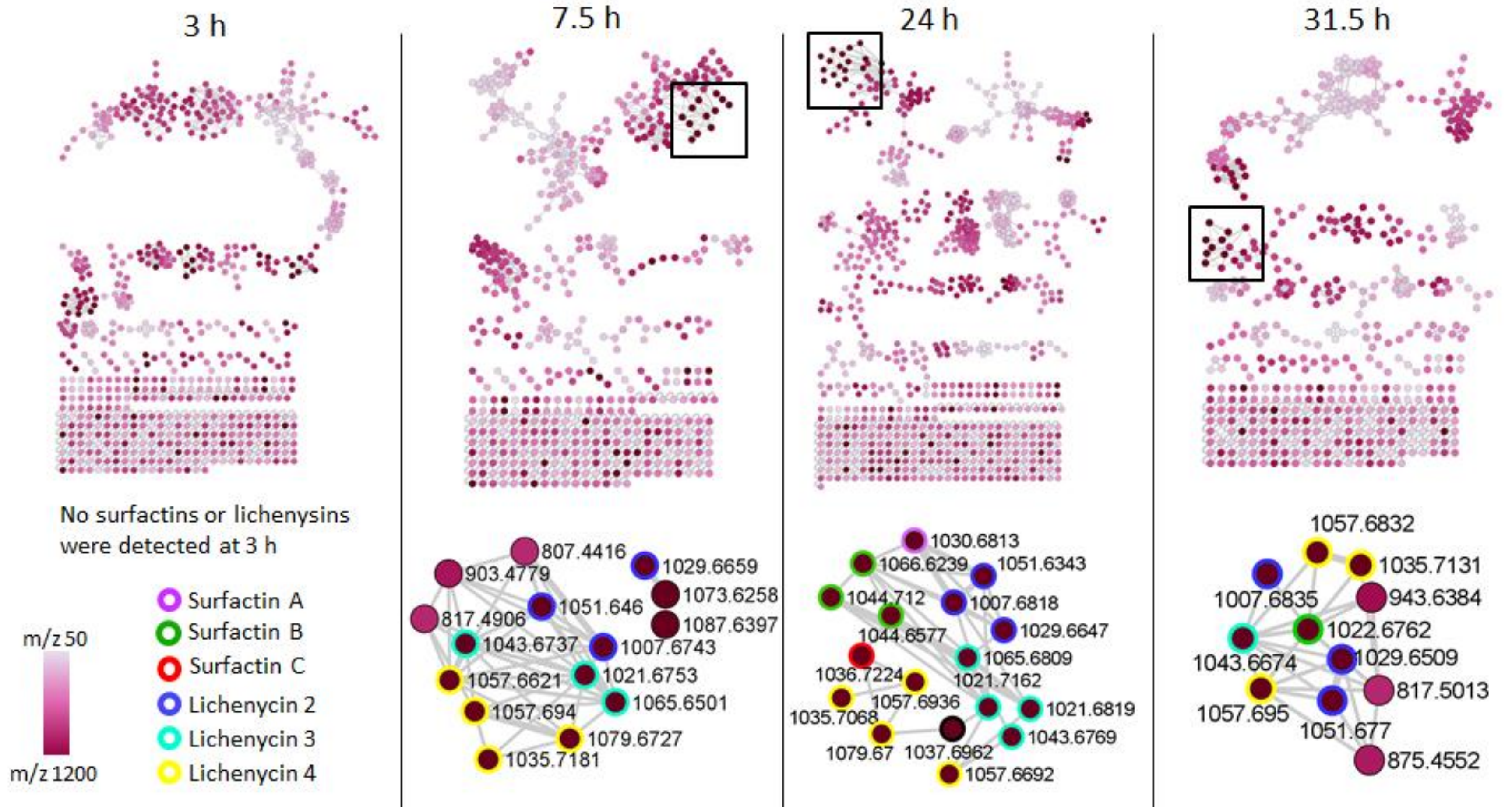

Figure S8. Molecular networking of positive electrospray ionisation (ESI$^+$) MS/MS spectra obtained from *B. licheniformis* 1001 over time. The zoom-in snaps shows that presence and type of lipopeptides varies depending on the bacterial growth phase. The stationary phase (24 h) showed more lichenysins. The nodes are coloured based on the acquired mass range (*m/z* 50-1200 Da) of the precursor ions: light pink nodes represent the smallest masses whereas the dark purple nodes represent the largest masses.

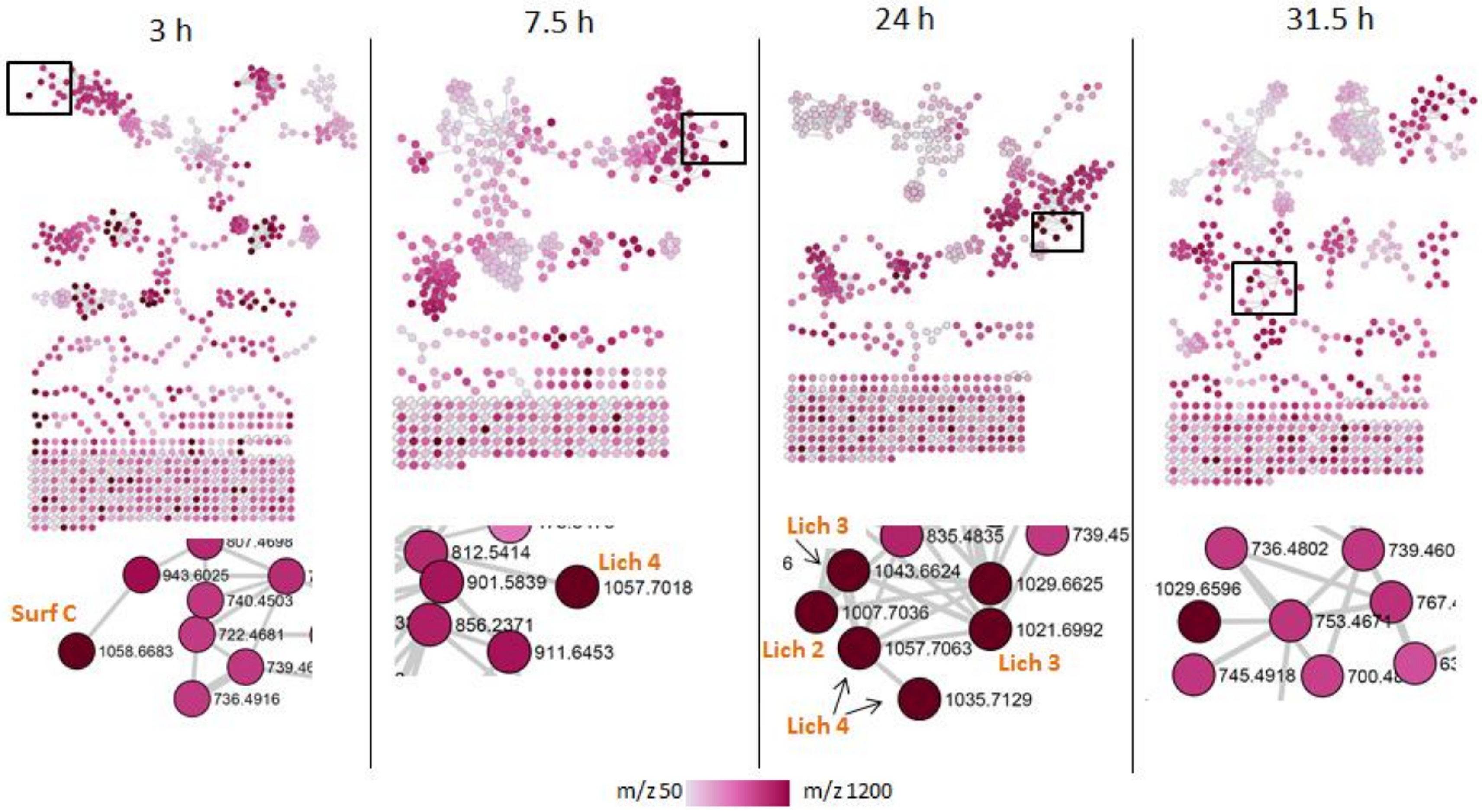

Figure S9. Molecular networking of positive electrospray ionisation (ESI$^+$) MS/MS spectra obtained from *B. licheniformis* M017 over time. The zoom-in snaps shows that presence and type of lipopeptides varies depending on the bacterial growth phase. The stationary phase (24 h) showed more lichenysins. The nodes are coloured based on the acquired mass range (*m/z* 50-1200 Da) of the precursor ions: light pink nodes represent the smallest masses whereas the dark purple nodes represent the largest masses. Surf = surfactin and Lich = lichenysin.
